# The role of human–pig interactions in modulating gut microbiota, stress, and performance

**DOI:** 10.1186/s40813-025-00465-2

**Published:** 2025-10-23

**Authors:** Lucas Venegas, Catalina Araya, Rocio Palomo, Nicolás Galarce, Daniela Siel, José Manuel Yáñez, Florencia Correa-Fiz, Javiera Calderón-Amor, Daniel Cartes, Maria Camila Ceballos, Agustín Piña, Sergio Guzmán-Pino, Daniela Luna

**Affiliations:** 1 Aquaculture Genomics Lab, Facultad de Ciencias Veterinarias y Pecuarias, Universidad de Chile, Av. Santa Rosa 11735, La Pintana, Santiago, 8820000 Chile; 2 Departamento de Ciencia Animal, Facultad de Ciencias Veterinarias y Pecuarias, Universidad de Chile, Av. Santa Rosa 11735, La Pintana, Santiago, 8820000 Chile; 3 Departamento de Medicina Preventiva Animal, Facultad de Ciencias Veterinarias y Pecuarias, Universidad de Chile, Av. Santa Rosa 11735, La Pintana, Santiago, 8820000 Chile; 4 Escuela de Medicina Veterinaria, Facultad de Medicina y Ciencias de la Salud, Universidad Mayor, Santiago, 8580745 Chile; 5 Unitat mixta d’Investigació IRTA-UAB en Sanitat Animal. Centre de Recerca en Sanitat Animal (CReSA). Campus de la Universitat Autònoma de Barcelona (UAB), Bellaterra, 08193 Catalonia Spain; 6 Escuela de Graduados, Facultad de Ciencias Veterinarias, Universidad Austral de Chile, Valdivia, 5090000 Chile; 7 Departamento de Ciencias Clínicas, Facultad de Ciencias Veterinarias y Pecuarias, Universidad de Chile, Av. Santa Rosa 11735, La Pintana, Santiago, 8820000 Chile; 8 Faculty of Veterinary Medicine, University of Calgary, 2500 University Drive NW, Calgary, AB T2N 1N4 Canada

**Keywords:** Pig microbiome, Human handling, Human–animal interaction, Hair cortisol, Pig welfare

## Abstract

**Background:**

The microbiota‒gut‒brain axis modulates pigs’ stress response, behavior, and overall welfare. Stressful management practices can disrupt gut microbiota (GM), negatively impacting pigs' health and welfare. This study evaluated how the quality of human handling influences stress-related physiological responses, productive performance, and gut microbiota (GM) composition in pigs during the nursery phase.

**Results:**

Female pigs (n= 36, 21 days old) were randomly assigned to three experimental groups (12 pigs/group, four pens per treatment): positive human handling (PHH), negative human handling (NHH), and a control group (CG). The PHH group experienced gentle tactile interactions, whereas the NHH group was subjected to chronic intermittent stress through acute stressors, and the CG group received minimal handling for routine practices. Hair cortisol concentrations were measured as an indicator of chronic stress (days 15 and 64). Productive performance was assessed through body weight (BW), average daily gain (ADG), average daily feed intake (ADFI), and feed conversion (FC). Fecal samples were collected at baseline (T0, day 16), mid-study (T1, day 37), and end of the study (T2, day 65) and analyzed using 16S rRNA gene amplicon sequencing to assess GM changes over time. Pigs in the PHH group showed a significant reduction in cortisol levels from baseline to post-treatment (P < 0.0001), while no significant changes were observed in the NHH group (P = 0.26). A smaller but significant decrease was also detected in the CG group (P = 0.001). PHH pigs had higher BW (P = 0.0009) and ADG (P = 0.03) during the later growth phase compared to NHH pigs. At T2, PHH pigs exhibited greater diversity and richness compared to NHH pigs, indicating a restorative effect on GM composition. Differential abundance analyses identified four bacterial genera that distinguished treatment groups: *Blautia*, *Megasphaera*, and *Subdoligranulum* were enriched in PHH pigs, while *Terrisporobacter* was enriched in NHH pigs. Additionally, bacterial interaction networks exhibited the least complex network in the NHH group, with ecological associations primarily involving *Clostridium* and *Terrisporobacter*.

**Conclusions:**

The quality of human handling influenced stress physiology, performance, and gut microbiota in pigs. Positive handling reduced cortisol levels, improved growth, and promoted microbial diversity, while negative handling was linked to decreased performance and reduced microbial network complexity. These results highlight the potential of positive interactions to enhance welfare and productivity, and identify specific bacterial genera as potential biomarkers differentiating negative and positive handling conditions.

**Supplementary Information:**

The online version contains supplementary material available at 10.1186/s40813-025-00465-2.

## Background

The quality of human‒animal relationship (HAR) is a critical determinant of farm animal welfare and has gained increasing attention in modern livestock systems. Its inclusion in the updated Five Domains Model of animal welfare highlights the recognition that interaction with humans can significantly influence animals’ affective states and overall welfare [1, 2].

The HAR is shaped by repeated interactions that animals learn to associate with positive, negative, or neutral experiences. These associations shape animals’ perception of human presence, modulating their behavioral and physiological responses accordingly [3, 4]. Negative interactions may trigger acute or chronic stress, characterized by activation of the autonomic nervous system, the hypothalamic-pituitary‒adrenal axis, and the immune system, ultimately compromising immune competence, growth, and overall health [2, 5, 6].

A growing body of evidence highlights the gut microbiota (GM) as a central biological mediator through which environmental stimuli, including stress, can influence animal health and welfare [7, 8]. The microbiota–gut–brain axis allows for bidirectional communication between the gut microbiome, the central nervous system, and immune function. Through this axis, stress-related signals can alter microbial communities, while microbial alterations can, in turn, affect brain function and behavior [9].

Stress-induced disruptions to the gut microbiome are frequently characterized by a reduction in microbial richness and diversity [10], along with a loss of potentially beneficial taxa when compared to unstressed individuals [11, 12]. Such changes are linked to impaired gut barrier function, increased inflammation, and disease susceptibility [13]. Chronic or repeated stressors, such as those encountered in routine production settings, e.g., weaning, mixing, transport, or handling, can produce lasting effects on the GM and negatively affect animal welfare and productivity [14–19].

Despite this, little is known about how different human handling procedures, ranging from gentle to aversive, may shape the gut microbial ecology of the gut via their influence on the stress response. This knowledge gap is particularly relevant given that human handling is an animal welfare influence factor, and, unlike many biological or environmental variables, it can be directly modified through practical strategies such as staff training [20]. Understanding whether and how human-animal relationship affects GM composition could therefore provide valuable insight into the biological mechanisms linking management practices to animal health, and potentially offer novel microbiome-based indicators for welfare assessment [21].

Based on this background, we hypothesized that different types of human–animal relationships —shaped by repeated interactions (positive, negative, or minimal)—can modulate stress responses and GM composition in nursery pigs, with implications for animal welfare and productivity. To test this hypothesis, we evaluated how varied human handling procedures influence stress-related physiological responses, productive performance, and GM profiles in pigs during the nursery period.

## Materials and methods

### Ethics statement

All experiments were conducted at the experimental pig facilities of the Facultad de Ciencias Veterinarias y Pecuarias (FAVET) at the Universidad de Chile (Santiago city, Chile, 34°21′ S, 71°18′ W) and were approved by the Institutional Animal Care and Use Committee of the University of Chile (Certificate No. 22552-VET-UCH-e1).

## Experimental procedure

### Animals and housing

A total of 36 female piglets, weaned at 21 d of age (weight 7.6 ± 1.1 kg) from 18 sows (PIC Genetics), were transported from a commercial pig farm to the experimental pig facility (day 1; Table 1). Upon arrival, each piglet was individually weighed. Animals were then allocated into 12 pens in groups of three, ensuring that no pen included more than one piglet from the same dam, while also maintaining similar body weight across pens as much as possible. This strategy aimed to reduce potential confounding due to maternal microbiota influence while preserving homogeneity in initial body weight. Once all animals were distributed in their designated pens, they were individually identified with numbered plastic ear tags. All pigs were housed in a nursery room equipped with automatic and forced ventilation and were exposed to identical environmental conditions throughout the study. The pens, measuring 1.20 × 2.0 × 0.9 m, were equipped with thick anti-fatigue rubber mats placed over concrete flooring. Each pen also included a heat lamp, a nursery feeder, and an independent water supply. The animals were fed a single standard commercial diet throughout the entire experimental period (Additional file 1). This diet was formulated to meet the nutritional requirements of pigs at weaning (5.5–7.5 kg), as specified by the PIC Nutrition and Feeding Guidelines [22], and provided nutrient levels sufficient to support growth during the nursery phase and the early growing phase. Although the diet was not adjusted as pigs gained weight, this decision was made to avoid introducing dietary variation as a potential confounding factor affecting gut microbiota composition. Additionally, because this study formed part of a larger research project, using a consistent diet ensured standardized conditions across all experimental groups and timepoints, supporting the comparability of microbiota-related outcomes.

Pigs had *ad libitum* access to feed and fresh water for the entire experimental period. They were exposed to auditory and olfactory stimuli from neighboring pens. However, visual and tactile contact between pens was prevented to avoid observational social learning effects, as previous studies have shown that pigs can form positive representations of humans by watching conspecifics being treated gently [23]. In this study, we aimed to generate positive associations with humans only in a specific treatment group; therefore, visual barriers were used to prevent pigs in the negative treatment group from seeing the positive handling applied to other pigs. All pigs were subjected to a period of acclimation to the facilities and the study personnel during the first 2 post-weaning weeks (days 1–14). Following the experimental procedures, all the animals were transported to an abattoir. Throughout the experiment, no animals exhibited clinical signs of disease; therefore, no antibiotics were administered. Furthermore, no pigs died or were removed from the study, and no diarrhea outbreaks were observed.

**Table 1:** Outline of the experiment

Day of the experiment	Pig age (d)	Event/Test	Place	Measures
1	21	Weaning	Commercial farm	-
1–14	21–34	Acclimation to experimental facility	Home pen	-
15	35	Hair shave	Experimental facility	Hair cortisol (baseline)
64	84	Hair reshave	Experimental facility	Hair cortisol
16–61	36–81	Human handling session	Home pen	-
16,37,65	36,57,85	Fecal sampling (T0, T1, T2)	Experimental facility	Microbiota
1,8,15,22,29,36,43,50,57,64	21,28,35,42,49,56,63,70,77,84	Productive performance	Experimental facility	BW, ADG, ADFI, FC

BW = Body Weight; ADG = Average Daily Gain; ADFI = Average Daily Feed Intake; FC = Feed Conversion

### Human handling treatments

The outline of all the experimental procedures is presented in Table 1. Following the acclimation period, each pen was randomly assigned to 1 of 3 handling treatments (4 pens/treatment; 3 pigs/pen) relative to different types of experience with humans over 45 d (days 16–61; Table 1). This study was part of a larger research project in which, in addition to what is reported here, we evaluated the behavior and nonlinear heart rate variability of the individuals (see Calderón-Amor et al. [2] for more details).

The handling protocols implemented were designed on the basis of previous studies to promote variations in fear-based or affiliative responses toward human interaction [5, 24–27], as follows.

### Positive human handling (PHH, N = 12 pigs)

Animals from four pens underwent long-term gentle tactile interaction. All handling procedures were performed inside the animals’ home pens; no pig was removed from its pen or separated from penmates during the interactions. Each pig received gentle contact for 2 min, handled individually but within the presence of its penmates, twice daily (from Monday to Friday) starting from the third week post-weaning, continuing for 6 weeks and 3 days (33 days total). The gentle handling protocol was standardized on the basis of Tallet et al. [25] and comprised the following steps: (1) the handler entered the home pen and remained standing still for 30 s; (2) subsequently, the handler proceeded to sit on a bucket, staying motionless for 1 min; (3) afterwards, the handler held out her hand toward the pig; if the pig did not move away, the handler tried to touch it; (4) if the pig was receptive to being touched, the handler began to gently stroke it with the palm of their hand, moving from the head to the back, at a rate of 1 stroke every 2 s for 2 min; (5) following this interaction with each pig, the handler proceeded to leave the pen.

### Negative human handling (NHH, N = 12 pigs)

Animals from 4 pens were subjected to long-term chronic intermittent stress through repeated exposure to various acute handling stressors [28]. As with the positive handling protocol, all procedures were conducted inside the home pens, and pigs remained in their social group during the handling sessions. Each pig was handled individually, while the other two pigs remained present in the pen. This approach allowed individual handling without removing animals from their social environment, thereby avoiding additional stress due to isolation or relocation. Pigs received various types of negative interactions, which were considered stressful but without being painful or causing evident injuries [24, 26] These were delivered to each pig for 2 min, with 1 pig at a time, twice daily (from Monday to Friday) for 5 weeks. The handling protocol was standardized with the handler entering the pen and implementing a combination of the following rough handling in random order: (1) Pursuing, capturing, and briefly lifting each pig using sudden and fast movements [26, 27]; and (2) attempting to place a rope around the snout of each pig [24]. The following 2 were excluded from the protocol in the second week of handling because the pigs exhibited behaviors suggestive of play, such as initiating a search for the ball to eat it, approaching the water cannon, or approaching the handler to explore it. They were: (3) Firing a rubber expandable ball (6 mm) or dart with a toy gun on the trunk of each pig. After that, if a pig entered the area around the handler, the handler shot the pig on the trunk with a ball while simultaneously moving her arms toward the pig [26, 27]. (4) Spray a jet of water onto the trunk of each pig using a toy gun.

This negative handling protocol was designed to induce chronic stress by repeatedly exposing animals to mildly aversive, unpredictable handling procedures over an extended period (weeks). This approach follows principles analogous to the chronic mild stress model commonly used in animal research, wherein animals are subjected to repeated, low-intensity stressors over extended periods, leading to cumulative effects on behavior and physiology, despite the transient nature of each individual stressor [28, 29].

###  Control group (CG, N = 12 pigs)

Pigs from 4 pens were subjected to minimal human contact, aligning with standard commercial practices for pig management, including feeding, pen cleaning and health assessments [23].

Throughout the experimental period, all animal handling was conducted exclusively by 3 trained female handlers. They wore distinctive coveralls (orange, blue, and gray) and black boots to facilitate human discrimination by pigs [30].

## Hair cortisol

### Hair cortisol sampling

Pigs’ hair samples were collected twice during the experimental period (Table 1) using the “shave-reshave” method [31]. A predefined bilateral area (10 × 15 cm) was shaved at the beginning of the study (day 15) to obtain baseline hair cortisol concentrations, and the same area was reshaven at the end of the treatment period (day 64) to collect regrown hair for post-treatment cortisol analysis. For each animal, approximately 250 mg of hair was collected from the dorsocaudal region [32], using the base of the tail as a reference point and ensuring proximity to the skin with an electric clipper, which was cleaned after each animal collection using 70% (v/v) ethanol to avoid cross-contamination. The shaving area’s localization was selected to minimize the risk of fecal or urine contamination. For sample collection, the animals were positioned on a surgical table, with their hindquarters resting on it and their forelimbs supported by an assistant’s shoulders. During the shaving process, one team member executed the shave while another maintained a rectangular template over the target area to ensure precision. Simultaneously, a third person placed a sheet of paper beneath the clipper to collect the trimmed hair. The samples were subsequently frozen at -20 °C inside hermetic plastic bags, avoiding exposure to light until further processing.

### Hair cortisol extraction

A schematic representation of the hair cortisol sample processing is shown in Additional file 2. The methodology for cortisol extraction was adapted from protocols described by Olvera-Maneu et al. 2021 [31] and Roelofs et al. 2019 [33]. Briefly, 250 mg of hair was weighed using a hermetic precision scale. Hair samples were washed 3 times with 5 mL of 99% (v/v) isopropanol and vortexed at 3,000 rpm for 2.5 min to remove external sources of steroids. The supernatant was subsequently separated by decantation. The samples were air-dried at room temperature over 5 d within a vertical laminar flow cabinet (Biobase Biotech Co., Ltd.). After drying, the samples were stored at -80 °C for 3 weeks. The samples were subsequently carefully sectioned using a scalpel, and 40 mg of hair per sample was collected into 2 mL microcentrifuge tubes. Four 2-mm zirconia balls were added to each tube before being placed in a ball mill (Tissuelyser II, Qiagen Benelux B.V., Antwerp, Belgium) at 2 × 15 min at 30 Hz. Once the process was completed, 1 mL of methanol was added to each sample, which was then incubated at 100 rpm and 36 °C for 24 h to facilitate corticosteroid extraction. After incubation, the samples were centrifuged at 9,500 rpm for 5 min, and 0.6 mL of the resulting supernatant was transferred to new tubes. The samples were dried in a vertical laminar flow cabinet until completely evaporated, then reconstituted with 0.25 mL of phosphate-buffered saline (PBS) buffer and vortexed at 1,200 rpm for 30 s. The reconstituted samples were subsequently stored at -20 °C until analyzed using the Salimetrics Salivary Cortisol ELISA Kit (Salimetrics, State College, Pennsylvania, USA), which was validated to measure hair cortisol concentrations accurately [34]. Sample analysis was performed with a microplate reader (EPOCH, BioTek^®^). All samples were assayed in duplicate, and their average was utilized for subsequent data analysis. Hair concentrations are expressed in pg/mg after conversion from µg/dL. The allocation of samples across plates was balanced according to the treatment of the pigs. The intra- and inter-assay coefficients of variation were 6.66% and 1.43% for Plate 1 and 7.6% and 2.52% for Plate 2, respectively.

For baseline cortisol analysis, hair samples were collected and processed individually from each pig (*n* = 36), following the procedure described above. After the extraction step, an equal volume of the reconstituted cortisol solution from each of the three pigs within the same pen was pooled to create a single representative sample per pen (*n* = 12 pens, 4 per treatment).

### Productive performance assessment

Productive performance was evaluated using four indicators: Body Weight (BW), Average Daily Gain (ADG), Average Daily Feed Intake (ADFI), and Feed Conversion (FC). Pigs were individually weighed once per week, from week 1 to week 10, using a calibrated digital scale. Daily feed intake (in grams) was evaluated per pen by recording the amount of feed offered. Feed refusals were collected and weighed weekly to estimate the feed consumption per pen.

Average Daily Gain (ADG) was determined by dividing the weekly body weight gain of each pig by the number of days in the interval. Average Daily Feed Intake (ADFI) was calculated by subtracting the feed refusals from the total feed offered each week and dividing the result by the number of pigs per pen and the number of days in the week. Feed Conversion (FC) was calculated as the ratio between ADG and ADFI, representing the efficiency of feed utilization over the productive period.

### Characterization of the gut microbiota

#### Fecal sample collection

Fecal samples were collected from each pig at three time points during the experimental period: days 16 (T0), 37 (T1), and 65 (T2). This sampling schedule was used to monitor temporal variations in GM composition in response to human interactions. Samples were collected from the rectum, taking advantage of the pigs’ natural defecation response following brief physical restraint of the animals. If defecation did not occur within the first 20 s, a disinfected thermometer was softly inserted into the rectum to stimulate defecation. All the fecal samples were collected using sterile disposable gloves and placed into sterile flasks. The samples were then promptly transported to the laboratory on ice and subsequently stored at -80 °C for further processing.

### DNA extraction

Microbial genomic DNA was extracted from 250 mg of each pig fecal sample using the QIAamp PowerFecal DNA Kit (Qiagen, Venlo, Netherlands), following the manufacturer’s instructions. The DNA concentration in each sample was determined using a Quantus™ fluorometer (Promega). Consistent with previous studies in pigs [14, 35], the hypervariable regions V3-V4 of the 16 S rRNA gene were amplified using the 341 F (5′-CCTAYGGGRBGCASCAG-3′) and 806R (5′-GGACTACNNGGGTATCTAAT-3′) primers following a 2-step PCR procedure. For the first PCR amplification (PCR1), Platinum II Taq Hot-Start DNA Polymerase was used with an initial denaturation step at 94 °C for 2 min, followed by 25 cycles at 94 °C for 15 s (denaturation), 56 °C for 15 s (annealing), 68 °C for 15 s (extension), and a final hold at 4 °C. A second PCR phase (PCR2) was conducted to incorporate barcodes and identify each sample, utilizing the Kapa HiFi Hot-Start mix with a Barcoding Cycle at 95 °C for 3 min, followed by 10 cycles at 95 °C for 30 s, 55 °C for 30 s, 72 °C for 30 s, 72 °C for 5 min, and a final hold at 4 °C. Quality control of the libraries was performed using the KAPA Library Quantification Kit (Roche Diagnostics Corporation, Indiana, USA). DNA sequencing was performed via Illumina MiSeq sequencing (250 paired-end) at McGill University and Génome Québec Innovation Center (Montreal, QC, Canada).

### Bioinformatics processing

The quality control of fastq paired-end sequences was assessed with FastQC software v. 0.11.9 [36] and handled with the Divisive Amplicon Denoising Algorithm (DADA2) v. 1.26.0 [37] package in R software v.4.2.3 [38]. Forward and reverse reads were truncated at 249 and 245 bp, respectively, ensuring a Phred quality score ≥ 20 and keeping the maximum expected errors set as default values (2 for both forward and reverse reads) with the filterAndTrim function. Filtered reads were then used for learning error rates (learnErrors function) and dereplication (derepFastq function) steps before inferring amplicon sequence variants (ASVs) with the dada2 function (“pool = pseudo” option). After ASV inference, paired-end sequences were merged using the mergePairs function. Chimera removal was performed with the removeBimeraDenovo function, and taxonomic classification of non-chimeric sequences was assigned using the RDP naive Bayesian classifier algorithm from the assignTaxonomy and addSpecies functions, utilizing the SILVA v.138 with a 99% identity criterion training set database [39]. The data were then combined into a phyloseq object using the phyloseq package v.1.42.0 [40].

### Microbial diversity analyses

Prior to the diversity analyses, the phyloseq object was filtered according to the following criteria: only taxa not assigned to the chloroplast, mitochondria, or Eukaryota were retained, as well as taxa detected > 4 times in > 20% of the samples. The samples were subsequently normalized (subsampled without replacement) to the lowest number of reads per sequence using the rarefy_even_depth function of phyloseq.

Alpha diversity estimators, including the Shannon index, Simpson index, Pielou’s evenness index and number of observed species, were obtained using the get_alphaindex function from the MicrobiotaProcess package [41]. To examine the variation in GM diversity between samples (beta diversity), Bray‒Curtis and Jaccard distances were calculated using the phyloseq distance function. Principal coordinate analysis (PCoA) was conducted on each distance matrix to visually represent dissimilarities in microbiota composition. Additionally, an abundance bar plot was generated to provide a comprehensive view of the microbial composition to depict the relative proportions of agglomerated taxonomic ranks.

### Differential abundance analysis between groups

To investigate the associations between the abundance of bacterial communities and human handling procedures, microbial differential abundance (DA) analyses were conducted using ANCOM-BC2 [42], Aldex2 [43], LEfSe [44], and MaAsLin2 [45] at each time point. The significance of the associations was determined using the corrected *P* -value (q) at the genus taxonomic level, with values < 0.05 considered statistically significant.

### Clustering and correlation network analyses

A hierarchical cluster analysis of the taxonomic composition of bacterial communities was generated with MicrobiomeAnalyst [46] using the Euclidean distance measure and the Ward clustering algorithm, considering normalized ASV counts with the total sum scaling (TSS) procedure.

Correlation network analysis considered ASVs count data from each handling procedure at the T2 sampling time point (day 65; *N* = 36) to estimate significant co-presence or mutual exclusion patterns using the CoNet tool V. 1.1.1. beta [47]. Four statistical tests (Pearson and Spearman rank correlations, Bray‒Curtis and Kullback‒Leibler nonparametric dissimilarity indices) were performed, considering a multiple similarity measures strategy following a distribution of all possible pairwise scores of ASV abundances. Then, thresholds for each measure were selected considering 1,000 positive and 1,000 negative edges to the initial network. The randomization and bootstrap scores steps of CoNet considered 1000 permutations for each edge and measure. The *P-*values obtained were merged using Brown’s method and corrected by Benjamini and Hochberg’s multiple hypothesis testing. Network characteristics were obtained using the NetworkAnalyzer tool and displayed on Cytoscape v. 3.9.1 [48] to visualize and compare the resulting networks.

### Statistical analysis

To evaluate the effects of treatment on cortisol concentrations, a linear mixed-effects model was fitted using the lmer() function in the *lme4* package in R [49]. Treatment (three levels: PHH, NHH, and CG) and sampling time (baseline and post-treatment) were included as fixed effects, along with their interaction. Pen was included as a random effect to account for clustering within groups. A total of 12 pens (*n* = 4 per treatment) were included in the analysis. Post hoc comparisons between baseline and post-treatment cortisol concentrations within each treatment were conducted using Tukey’s (HSD) test, as implemented in the *emmeans* package. Model assumptions of normality and homogeneity of variances were assessed and met.

For both body weight and average daily gain, linear mixed-effects models were fitted using the lmer() function from the lme4 package [49]. The models included treatment, period, and their interaction as fixed effects, with individual pigs nested within the pen as a random effect. Period corresponded to weeks of measurement for body weight (wk 1, 3, 6, 10), and to intervals between weeks (1–3, 3–6, 6–10) for average daily gain. Post hoc comparisons were performed using estimated marginal means via the emmeans package [50].

For both average daily feed intake and feed conversion ratio, linear models were used with treatment, period, and their interaction as fixed effects, and pen as the statistical unit. Measurements were collected at the pen level across the same periods (1–3, 3–6, and 6–10 weeks). The models were fitted using the lm() function in R. When significant effects were detected, post hoc comparisons were conducted using estimated marginal means with the emmeans package.

Linear mixed models were applied using the lmer() function from the lme4 package [49] to assess the relationships between explanatory variables and microbiota alpha diversity parameters. The effects of the explanatory variables were assessed using Type II Wald chi-square tests from the car package [51]. When there were significant effects, post hoc comparisons were made using Tukey’s adjustments with the emmeans package [50]. Visual inspection of residuals was conducted to check for normality and homogeneity of variances across all models.

As an example, the models for microbiota parameters were structured as follows: Microbiota parameter (e.g., Shannon) ∼ Time + Treatment + Time×Treatment + (1|Pen/Pig). The results are presented as the mean and standard error of the mean (SEM).

Beta diversity differences were evaluated using a permutational multivariate analysis of variance (PERMANOVA) with 1,000 permutations, using the adonis function of the vegan package v.2.6.4 [52] comparing treatment groups by each time. Alpha and beta diversity results were plotted using the plotly package v. 4.10.4 [53] and ggplot2 package [54] in R. Significance was set at *P* < 0.05 for all analyses. All statistical analyses were performed using R software version 4.2.3 [38].

## Results

### Hair cortisol concentration

The estimated cortisol concentrations marginal means (± SE) for baseline and post-treatment, respectively, were 16.33 ± 1.36 pg/mg, and 10.90 ± 1.36 pg/mg in the CG group; 23.85 ± 1.36 pg/mg and 8.68 ± 1.36 pg/mg in the PHH group; and 12.69 ± 1.36 pg/mg and 14.47 ± 1.36 pg/mg in the NHH group.

**Fig. 1:**
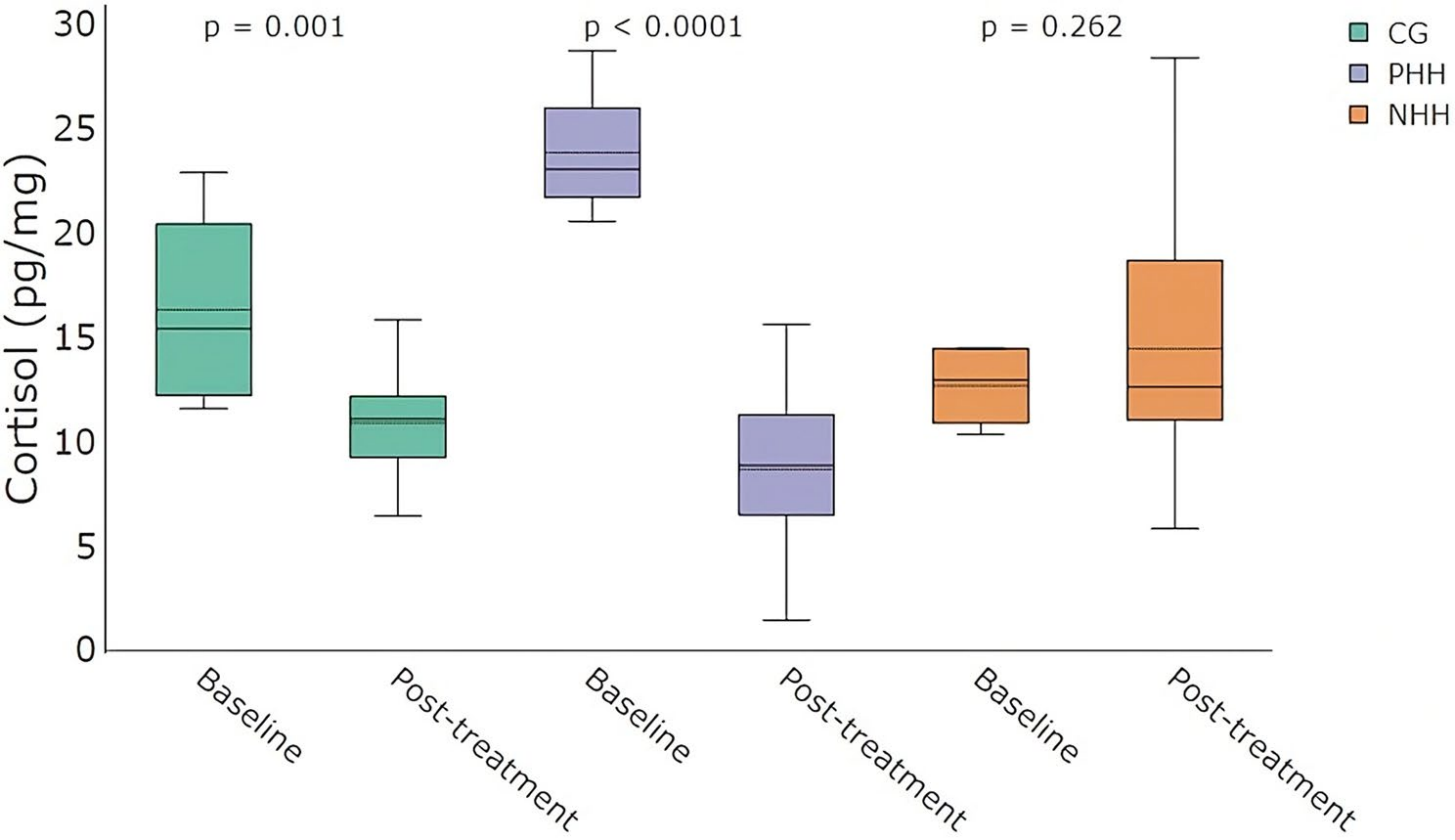
Boxplots of hair cortisol concentrations at baseline and post-treatment across the three handling treatments: control group (CG), positive human handling (PHH), and negative human handling (NHH). Each boxplot shows the median (solid horizontal line) and the mean (dashed line). P-values above each group indicate results from Tukey’s post hoc comparisons between baseline and post-treatment within each treatment group

There was a significant interaction between sampling time and treatment on hair cortisol concentrations (*F*(2, 57) = 29.42, *p* < 0.001). Post hoc comparisons showed a significant decrease in cortisol concentrations from baseline to post-treatment in the PHH group (estimate = − 15.17 ± 1.57 pg/mg, *p* < 0.0001), and in the CG group (estimate = − 5.43 ± 1.57 pg/mg, *p* = 0.0010), while no difference was observed in the NHH group (estimate = 1.78 ± 1.57 pg/mg, *p* = 0.2616) (Fig. 1).

### Productive performance

#### Body weight

Body weight increased over time across all treatment groups (*F*(3, 99) = 1173.54, *P* < 0.001). There was no main effect of treatment on body weight (*F*(2, 9) = 2.24, *P* = 0.16). However, the interaction between treatment and period was significant (*F*(6, 99) = 4.69, *P* = 0.0003). Post hoc comparisons revealed that, at week 10 (BW10), pigs in the PHH group (29,669 ± 719 g) were heavier than those in both the CG group (26,318 ± 719 g; *P* = 0.01) and the NHH group (25,210 ± 719 g; *P* = 0.0009). No significant differences in BW were observed between treatments at week 1, 3, or 6 (*P* > 0.05).

#### Average daily gain

Average daily gain increased progressively over time across all treatment groups (*F*(2, 66) = 321.69, *P* < 0.001). Treatment affected ADG (*F*(2, 9) = 4.59, *P* = 0.044), and the interaction between treatment and period approached significance (*F*(4, 66) = 2.41, *P* = 0.058).

Post hoc comparison for the main effect of treatment showed that pigs in the positive handling group (335 ± 15 g/day) had higher overall ADG compared to the negative handling group (273 ± 15 g/day, *P* = 0.03). No significant differences were observed between PHH and the CG group (CG; 292 ± 15 g/day, *P* = 0.16), nor between CG and NHH (*P* = 0.64).

Post hoc comparisons of the interaction revealed that treatment effects emerged only in the final growth phase (week 6–10). In this period, pigs in the positive handling group (507 ± 18.4 g/day) had significantly higher ADG than pigs in both the NHH group (409 ± 18.4 g/day; *P* = 0.003) and the CG group (424 ± 18.4 g/day; *P* = 0.01). No significant differences between treatments were observed in wk1–3 or wk3–6 (*P* > 0.05).

### Average daily feed intake

Feed intake increased significantly across the three measured phases (*F*(2, 27) = 66.90, *P* < 0.001). The average daily feed intake was significantly higher in the second phase (wk3–6; 554 ± 33.2 g/day) compared to the first (wk1–3; 299 ± 33.2 g/day, *P* < 0.0001), and highest in the final phase (wk6–10; 842 ± 33.2 g/day), which differed significantly from both previous phases (all *P* < 0.0001). Neither the main effect of treatment (*F*(2, 27) = 2.68, *P* = 0.09) nor the treatment × period interaction (*F*(4, 27) = 0.40, *P* = 0.81) were statistically significant.

### Feed conversion

Feed conversion ratio varied significantly across phases (F(2, 27) = 7.15, *P* = 0.003). Estimated marginal means showed that feed conversion was the highest during the first period (wk1–3: 0.610 ± 0.0187), and significantly lower in the second (wk3–6: 0.513 ± 0.0187, *P* = 0.003) and third periods (wk6–10: 0.539 ± 0.0187, *P* = 0.03). There was no difference between wk3–6 and wk6–10 (*P* = 0.60). Neither treatment (*P* = 0.61) nor the treatment × period interaction (*P* = 0.68) had an effect on feed conversion.

### Microbiota analysis

#### Richness, diversity, and composition of the fecal microbiota

A total of 8,763,469 sequences were obtained from 108 pig samples collected at time points T0, T1 and T2. Following taxonomic assignment, filtering and exclusion of taxa (“Chloroplast”, “Mitochondria”, and “Eukaryota”), 3,289 ASVs were identified and distributed within bacterial (3,286 ASVs, 99.909%) and archaeal (3 ASVs, 0.091%) taxa. The lowest number of sequences per sample was 3,479, and the highest was 56,469. Finally, normalization procedures retained the lowest library size per sample (3,479), comprising 375,732 reads across all the samples.

From a taxonomic perspective, 5 phyla were identified across all the samples, with *Bacillota *(89.3%) and *Bacteroidota* (10.5%) accounting for > 99% of the identified bacterial community. Additionally, *Patescibacteria* (0.072%), *Proteobacteria* (0.071%), and *Euryarchaeota* (0.026%) were also detected, although they represented a considerably lower proportion of the community (Additional file 3). At the family level, 23 families were identified, with *Clostridiaceae* (37.75%), *Lactobacillaceae* (16.12%), and *Lachnospiraceae* (12.74%) being the most predominant. At the genus level, 65 genera were identified; the majority belonged to *Clostridium sensu stricto 1* (36.84%), *Lactobacillus* (11.88%), *Terrisporobacter* (7.79%), and *Prevotella_9* (7.19%). The taxonomic composition at the genera level for each sampling time and treatment is reported in Additional file 4, whereas detailed results of the top 10 genera associated with the PHH, NHH and CG groups for each sampling time are provided in Additional file 3.

**Fig. 2:**
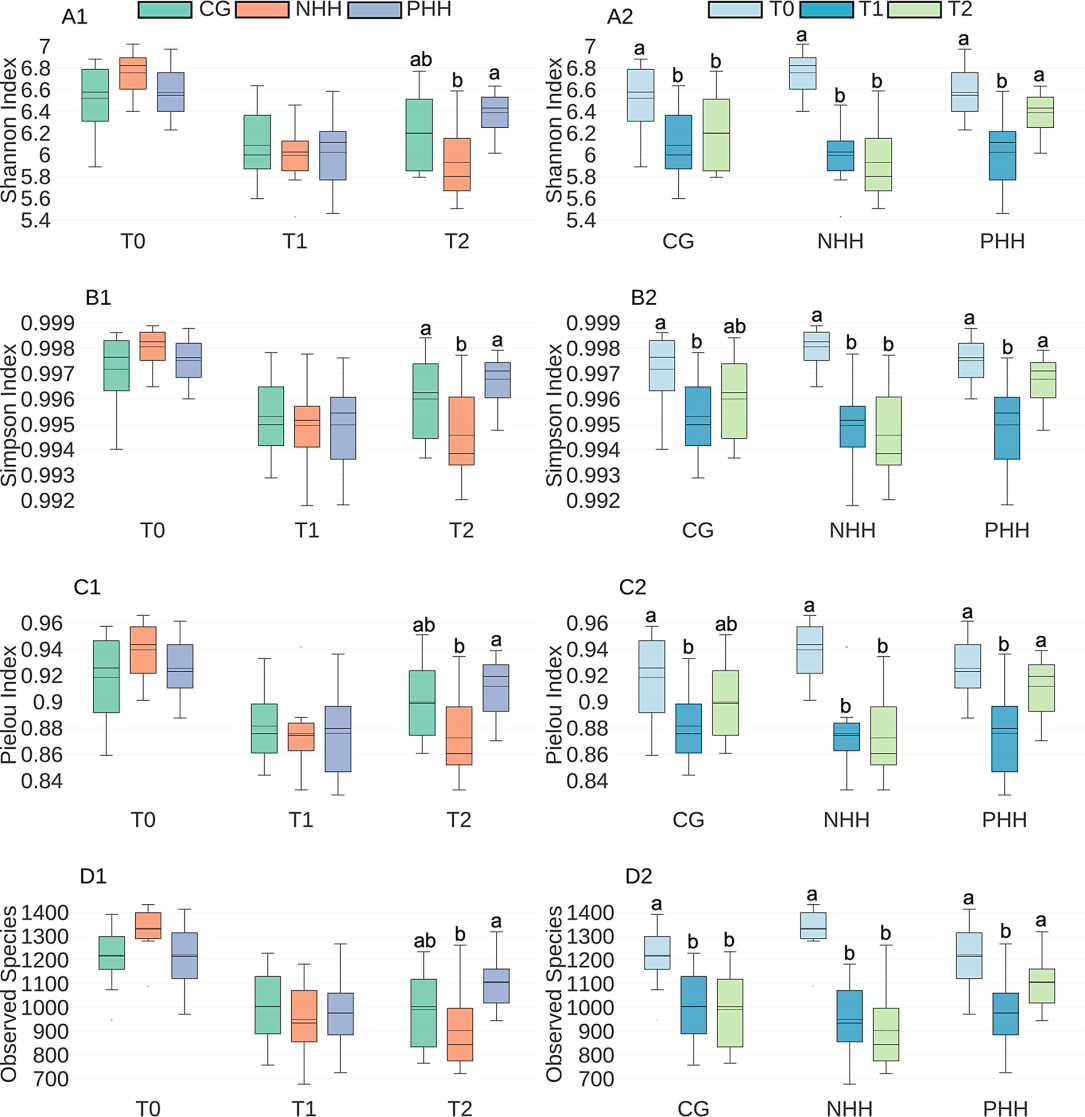
Boxplots of alpha-diversity metrics of bacterial communities in pigs under different human handling over time**.** Pigs’ fecal samples were analyzed between and within (temporal changes) treatment groups (PHH, NHH and CG) at 3 sampling periods (T0, T1, and T2). (**A1**) Shannon index between treatments; (**A2**) Shannon index within treatments; (**B1**) Simpson index between treatments; (**B2**) Simpson index within treatments; (**C1**) Pielou’s evenness index between treatments; (**C2**) Pielou’s evenness index within treatments; (**D1**) Observed Species index between treatments; and (**D2**) Observed Species index within treatments. Continuous horizontal lines within boxes represent the median (──), whereas dashed lines represent the mean (----). The tops and bottoms of the boxes represent the 75th and 25th quartiles, respectively. Bars without common superscripts indicate statistically significant differences (Linear mixed models, *P*-value < 0.05 and Tukey post hoc test). CG = control group; NHH = negative human handling; PHH = positive human handling. T0 = day 16 (baseline); T1 = day 37; T2 = day 65

The impacts of human handling on the alpha-diversity metrics of bacterial communities in pigs’ fecal samples between and within treatment groups at various sampling periods are depicted in Fig. 2. The results from the linear mixed models and post hoc analyses are shown in Additional files 5 and 6, respectively. Descriptive statistics for treatment and time effects are summarized in Additional file 7.

Regarding the Shannon index, there was an interaction effect between time and treatment (X² = 23.20, *P* = 0.0001). At T2, PHH exhibited a higher Shannon diversity compared to NHH (*P* = 0.001) (Fig. 2A1). When changes within treatments were examined, both CG and NHH had a consistent decline in Shannon diversity from T0 to T1 (CG: *P* = 0.0002; NHH: *P* < 0.0001) and from T0 to T2 (CG: *P* = 0.008; NHH: *P* < 0.0001). In contrast, the PHH group followed a different trend; although there was a decrease from T0 to T1 (*P* < 0.0001), the diversity increased from T1 to T2 (*P* = 0.002) (Fig. 2A2).

Similarly, for the Simpson index, there was an interaction effect between time and treatment (X² = 19.58, *P* = 0.0006). At T2, both the CG (*P* = 0.04) and the PHH (*P* = 0.001) presented higher Simpson scores than did the NHH (Fig. 2B1). Within treatments, CG presented a decrease in Simpson scores from T0 to T1 (*P* = 0.001), whereas NHH displayed a consistent decrease from T0 to T1 (*P* < 0.0001) and T2 (*P* < 0.0001). The PHH group followed a different pattern; although the Simpson scores decreased from T0 to T1 (*P* < 0.0001), it increased from T1 to T2 (*P* = 0.002) (Fig. 2B2).

Following the same pattern, Pielou’s evenness analysis revealed an interaction effect between time and treatment (X² = 20.17, *P* = 0.0004). At T2, the PHH had higher Pielou’s scores compared to NHH (*P* = 0.004) (Fig. 2C1). Within the treatments, the CG exhibited a decrease in Pielou’s score from T0 to T1 (*p* = 0.0009), whereas the NHH consistently decreased from T0 to T1 (*P* < 0.0001) and T2 (*P* < 0.0001). The PHH group followed a different trajectory; although there was a decrease from T0 to T1 (*P* < 0.0001), Pielou’s score increased from T1 to T2 (*P* = 0.001) (Fig. 2C2). Lastly, regarding the observed species, an interaction effect between time and treatment was detected (X² = 20.55, *P* = 0.0003). At T2, PHH had more observed species compared to NHH (*P* = 0.002) (Fig. 2D1). Within treatments, both CG and NHH exhibited a consistent decline in observed species from T0 to T1 (CG: *P* = 0.0004; NHH: *P* < 0.0001) and from T0 to T2 (CG: *P* = 0.0001; NHH: *P* < 0.0001). The PHH group followed a different pattern; although there was a decrease from T0 to T1 (*P* < 0.0001), the number of observed species increased from T1 to T2 (*P* = 0.03) (Fig. 2D2).

Analysis of beta-diversity metrics, specifically through PCoA based on Bray‒Curtis and Jaccard dissimilarity matrices (Additional file 8 and Additional file 9, respectively), revealed that the bacterial communities between PHH and NHH groups differed only at T2, according to the Bray‒Curtis (PERMANOVA, R^2^ = 0.125, *P* = 0.036) and Jaccard dissimilarity indices (PERMANOVA, R^2^ = 0.009, *P* = 0.027) (Table 2). However, distinction using the PERMANOVA test was not supported for the CG, which did not differ from the NHH or PHH groups (*P* > 0.05).

**Table 2:** PERMANOVA of fecal bacterial communities from pigs exposed to different human handling over time

Time	Compared groups		Permanova Bray‒Curtis dissimilarity (adjusted *P*-value)	Permanova Jaccard dissimilarity (adjusted *P*-value)
T0	CG	NHH	0.882	0.588
	CG	PHH	1.000	0.858
	NHH	PHH	0.867	0.288
T1	CG	NHH	1.000	0.273
	CG	PHH	1.000	1.000
	NHH	PHH	1.000	0.111
T2	CG	NHH	0.147	0.336
	CG	PHH	0.729	0.117
	NHH	PHH	0.036*	0.027*

PERMANOVA results are based on Bray‒Curtis and Jaccard dissimilarity matrices. CG = control group; NHH = negative human handling; PHH = positive human handling; T0 = day 16 (baseline); T1 = day 37; T2 = day 65. *Different (*P* < 0.05)

### Microbial differential abundance

A set of differential abundance (DA) tests, including ANCOM-BC2, Aldex2, LEfSe, and MaAsLin2, was performed to identify differences in the abundances of individual taxa among the CG, NHH, and PHH groups at T0, T1, and T2. Genera detected by at least one DA test across handling treatments and time points (*P* < 0.05) are described in Additional file 10. A total of 15 and 17 genera were detected by a single DA test at T0 and T1, respectively. When the PHH and NHH groups were compared with the CG reference, there was no difference in taxa abundance at T2 (*P* > 0.05). However, eight genera were detected by at least one DA test when comparing the PHH to the NHH group (Table 3). We considered differentially abundant taxa when they were detected with at least 2 statistical tests (score *≥* 2), resulting in the identification of four taxa: *Blautia*, *Megasphaera, Subdoligranulum*, and *Terrisporobacter* (Additional file 11). Thus, *Terrisporobacter* (10.79 vs. 6.54%), were enriched in the NHH group compared with the PHH group, whereas *Blautia* (4.00 vs. 1.57%), *Megasphaera* (4.00 vs. 1.20%), and * Subdoligranulum* (2.29 vs. 1.03%) were differentially increased in pigs positively handled compared with those negatively manipulated at T2 (Table 3).

**Table 3:** Scoring table of the four DA tests comparing the genus abundances between the PHH and NHH groups at T2

Differential abundance (DA) tests					
Genus	ANCOM-BC2^a^	Aldex2^b^	LEfSe^c^	MaAsLin2^d^	Score
*Blautia*	(-)	(-)	(+)	(+)	2
*Clostridium sensu stricto 1*	(-)	(+)	(-)	(-)	1
*[Eubacterium] halii group*	(-)	(-)	(-)	(+)	1
*Megasphaera*	(-)	(+)	(+)	(-)	2
*Prevotella_9*	(-)	(-)	(+)	(-)	1
*Subdoligranulum*	(-)	(-)	(+)	(+)	2
*Terrisporobacter*	(+)	(+)	(-)	(-)	2
*UCG-005*	(-)	(+)	(-)	(-)	1

The score values represent the number of genera significantly differentiated by each of the 4 methods (ANCOM-BC2, Aldex2, LEfSe and MaAsLin2). A genus was considered differentially abundant when the score was 2 or higher. ^a^ ANCOM-BC2: *Terrisporobacter* (q = 0.024); ^b^ Aldex2: *Clostridium sensu stricto 1* (Welch’s t test = 0.014; Wilcoxon test = 0.015), *Terrisporobacter* (Welch’s t test = 0.003; Wilcoxon test = 0.006), *Megasphaera* (Welch’s t test = 0.065; Wilcoxon test = 0.014), and *UCG-005* (Welch’s t test = 0.014; Wilcoxon test = 0.008). ^c^ LEfSe: *Subdoligranulum* (LDA score = 2.066), *Blautia* (LDA score = 2.311), *Megasphaera* (LDA score = 2.326), and *Prevotella_9* (LDA score = 2.355). ^d^ MaAsLin2: *Blautia* (q = 0.037), *Subdoligranulum* (q = 0.037), and *[Eubacterium] halli group* (q = 0.037)

### Hierarchical cluster analyses

**Fig. 3:**
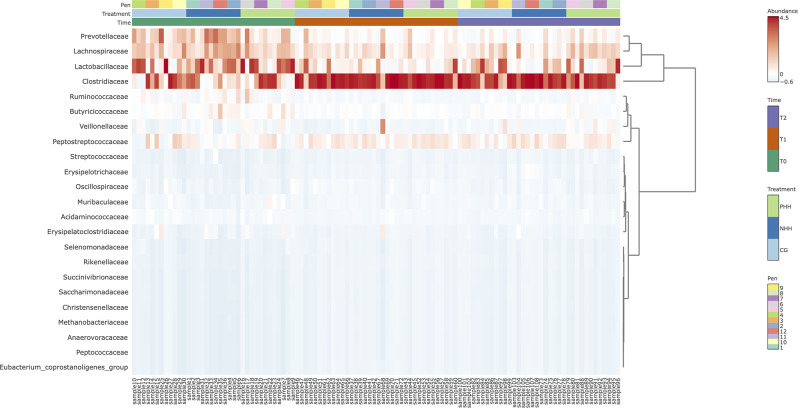
Hierarchical clustering of bacterial communities in pigs under different human handling over time (at the family level). Hierarchical cluster analysis was generated by MicrobiomeAnalyst package, using Euclidean distance measure and the Ward clustering algorithm. The color range represents the abundance of standardized samples. CG = control group; NHH = negative human handling; PHH = positive human handling. T0 = day 16 (baseline); T1 = day 37; T2 = day 65

**Fig. 4:**
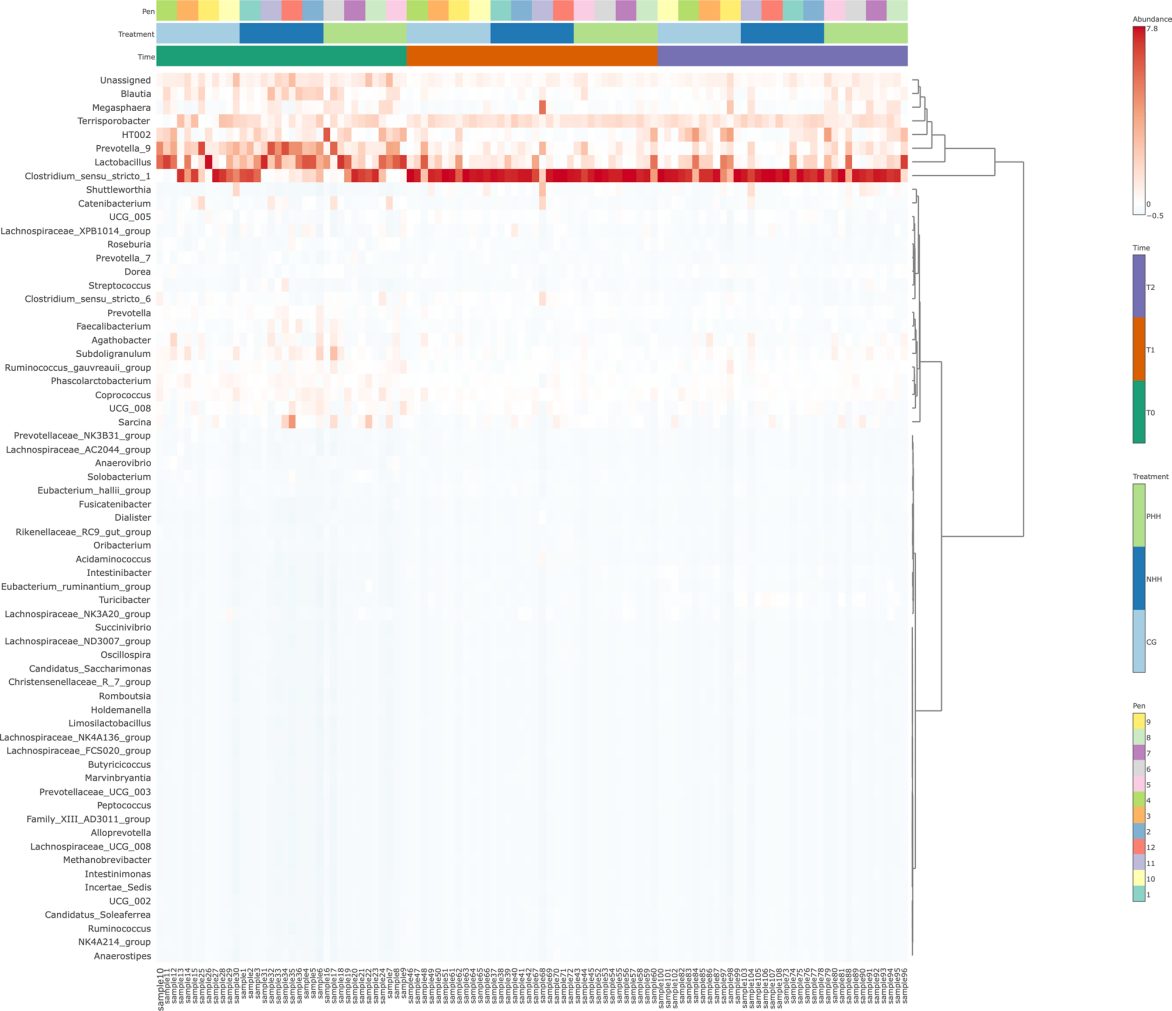
Hierarchical clustering of bacterial communities in pigs under different human handling over time (genus level). Hierarchical cluster analysis was generated by MicrobiomeAnalyst package, using Euclidean distance measure and the Ward clustering algorithm. The color range represents the abundance of standardized samples. CG = control group; NHH = negative human handling; PHH = positive human handling. T0 = day 16 (baseline); T1 = day 37; T2 = day 65

To characterize bacterial taxonomy over time and across treatment groups, hierarchical cluster analyses were conducted at the family and genus levels, as depicted in Figs. 3 and 4, respectively. These analyses resulted in the classification of samples into 2 primary clades, with the top clade representing the most abundant taxa among the standardized samples. At T0, all the samples exhibited a high degree of taxonomic variability, regardless of the handling procedure. This initial diversity was followed by a remarkable increase in the abundance of the *Clostridiaceae* family and the *Clostridium* genus across all groups by T1. By T2, a resurgence of certain taxonomic groups, notably *Lactobacillus*, was observed, which was particularly pronounced in the CG and PHH groups.

### Interaction network analysis of bacterial communities of pigs subjected to different human handling

**Fig. 5:**
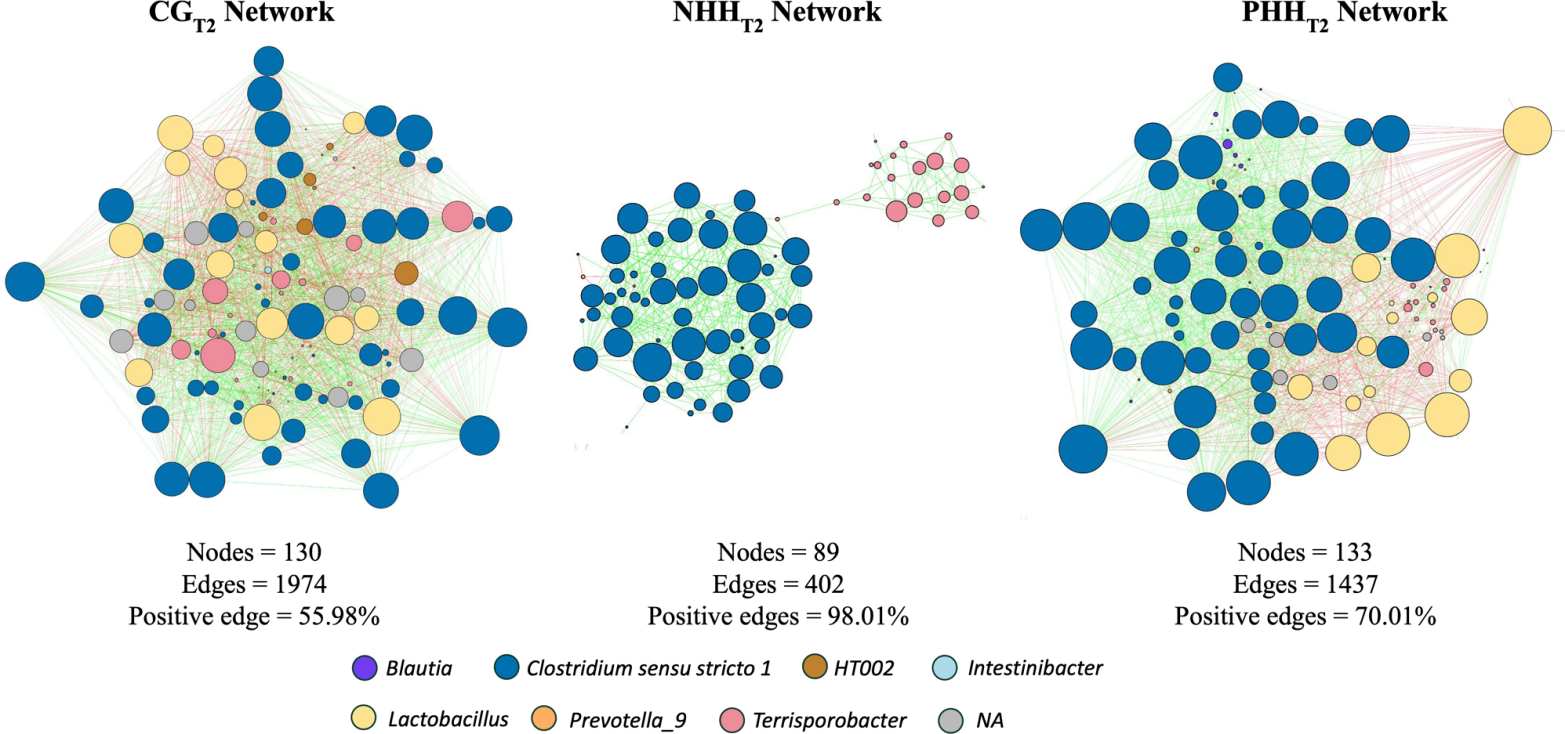
Microbial interaction networks of bacterial communities present in fecal samples from pigs at T2. Four similarity measures (Bray–Curtis and Kullback–Leibler dissimilarity, Pearson, and Spearman correlation) were estimated on the basis of the ASVs count. Each node and edge represent an ASV and a significant pairwise association between them (green lines = positive correlations; red lines = negative correlations), respectively. Node colors represent distinct genera. The node size represents the degree of interactions. CG = control group; NHH = negative human handling; PHH = positive human handling; Positive edges = percentage of positive edges from total edges

Microbial association networks revealed differences in response to distinct handling procedures in pigs, particularly regarding the number of nodes, edges, and the percentage of positive edges observed at T2, between the NHH group compared to the CG and PHH groups (Fig. 5). The interaction network for the CG group consisted of 130 nodes interconnected by 1974 edges, 55.98% of which were positive edges. The PHH group’s network comprised 133 nodes linked by 1437 edges, with a greater proportion of positive edges (70.01%). The NHH group network was characterized by the lowest number of nodes and edges, with 89 nodes and 402 edges and a high percentage of positive edges (98.01%) (Additional file 12). The degree of nodes differed among all treatments at T2 (*P* < 0.05) when all connections and specifically negative connections with other nodes were considered. However, positive connections differed only when the CG and NHH groups were compared (*P* < 0.05) (Additional file 13).

When network topology parameters at T2 were examined, network complexity decreased, with groups ranking in descending order of complexity as follows: CG > PHH > NHH. Compared with the CG and PHH groups, the NHH group presented a lower average clustering coefficient and network density. Similarly, the NHH group presented a lower node degree of negative connections compared to CG and PHH groups (Fig. 5; Additional file 13).

With respect to the NHH network, the genera *Clostridium sensu strictu 1* and *Terrisporobacter* emerged as unique interconnected taxa, concentrating the positive interaction ratio between them (Fig. 5). The *Lactobacillus* genus was also observed in the CG and PHH networks but was absent in the NHH network, which was attributed to the predominance of *Clostridium sensu stricto 1*, which established a negative correlation with *Lactobacillus*. Overall, *Lactobacillus* and *Clostridium* genera were relevant taxa that varied in abundance and community structure, demonstrating specific responses to the different handling procedures over time.

## Discussion

The swine GM composition plays a crucial role in the host’s physiology, immunity, and welfare [55]. As social animals, pigs naturally interact with humans and can develop positive relationships with their caretakers [56]. While positive handling can enhance welfare and productivity, pigs may still be exposed to negative human interactions, such as rough handling, unpredictable management, or loud noises, particularly in systems where staff training, supervision, or welfare protocols are limited. Such interactions can increase fear of humans and induce acute or chronic stress [5, 24, 57], potentially influencing GM composition and promoting intestinal dysbiosis. In this study, we investigated the effects of human handling quality on GM composition, stress-related physiological markers, and productive performance in pigs. While negative handling was associated with reduced microbial diversity and less complex microbial networks, post-treatment hair cortisol concentrations of pigs exposed to negative handling did not differ from their baseline levels. In contrast, pigs subjected to positive handling exhibited a reduction in cortisol concentrations over time, suggesting a stress-buffering effect of positive interactions with humans. Regarding productive performance, all pigs had an expected growth over time; however, those subjected to positive handling achieved superior body weight and daily gain during the later growth phase. No consistent effects of handling were observed on feed intake or feed conversion ratio. These results suggest that the quality of human-animal relationship influences both microbial and productive parameters in pigs.

### Hair cortisol as an indicator of chronic stress

Hair cortisol concentrations revealed distinct physiological responses among treatments. Cortisol concentrations decreased in the PHH and CG groups over time, while the NHH group showed no significant change relative to baseline. Contrary to expectations, negative handling did not induce elevated cortisol levels, and post-treatment concentrations remained generally low. These results are consistent with Carreras et al. [58], who also reported no differences in hair cortisol concentrations in fattening pigs exposed to aversive human interactions, such as immobilization, exposure to water, and loud noises. It is plausible that the intensity, duration, or frequency of the stressors applied in the NHH group were insufficient to generate a sustained activation of the hypothalamic–pituitary–adrenal (HPA) axis detectable through hair cortisol. Alternatively, these results may reflect a limitation of hair cortisol as a biomarker, particularly for detecting moderate or intermittent stress exposure in pigs, such as those applied in this study. Indeed, cortisol accumulation in hair is influenced by several individual and contextual factors, such as individual variability, genetic background, housing conditions, and social dynamics, that can affect its sensitivity and interpretation [32, 59]. As such, hair cortisol may fail to capture more nuanced or early biological responses to chronic stress.

Conversely, pigs exposed to positive handling exhibited a marked decrease in hair cortisol, consistent with studies demonstrating that affiliative human-animal interactions can buffer stress responses in several species [60–62]. For example, Coppola et al. [61] revealed that shelter-housed dogs, typically exposed to stressful environments, showed decreased salivary cortisol levels after receiving daily 45-minute sessions of positive tactile and verbal interaction. In pigs, gentle tactile interactions have been linked to positive emotional states and may serve as a buffer against stress through mechanisms involving social support [63, 64]. One proposed pathway involves the release of oxytocin during affiliative contact, which is related to stress response modulation [65]. Oxytocin can attenuate the HPA axis activity and reduce cortisol concentrations in both humans and animals [65–67]. Therefore, it is plausible that repeated exposure to gentle handling and affiliative interactions over the six-week period led to physiological downregulation of the HPA axis in the PHH group, contributing to the lower cortisol accumulation in hair.

Interestingly, the CG group also exhibited a significant, yet more modest, reduction in hair cortisol concentrations over time. This may be explained by the consistent, non-aversive human presence during routine cleaning tasks, which involved the stockperson entering the pen without applying direct tactile interaction. Although this type of exposure lacked the intentional positive reinforcement provided in the PHH group, it may have allowed pigs to habituate to human presence and perceive it as neutral or non-threatening. Previous studies support this interpretation, for example, Brajon et al. [26] reported that piglets exposed to a passive human presence, even without direct contact, developed more positive affiliative behaviors toward humans and presented reduced fear behavioral signs. Calderón-Amor et al. [68] similarly reported that broiler chicks exposed to a static human during conditioning later had reduced fear responses, spending more time in proximity to the human compared to controls, despite the absence of direct interaction. Therefore, the decrease in cortisol observed in the CG group might reflect a moderate form of habituation-based stress buffering. This observation aligns with the notion that not only the quality but also the consistency and predictability of human exposure can shape animals’ stress responses, even in the absence of explicit positive interactions. Thus, while the cortisol reduction in the CG group was less pronounced than in the PHH group, it suggests that familiar, predictable human presence may still contribute to stress mitigation in pigs [2].

### Productive performance

Both BW and ADG were higher in the pigs subjected to positive human handling compared to those in the control and negative handling groups. These differences emerged in the later phase of the experiment, specifically at week 10 for BW (BW10) and during weeks 6 to 10 (wk6-10) for ADG.

The effect of gentle or pleasant handling on productive parameters has been previously reported in several species, including poultry [69], dairy calves [70], and pigs [24, 71, 72]. In broiler chickens, repeated gentle stroking over three weeks was associated with increases in BW and improved feed conversion ratio by day 46 [69]. Similarly, Lürzel et al. [70] observed an increase in ADG in stroked dairy calves compared to controls over a two-week period.

Our results are consistent with Hemsworth et al. [24], who reported increased ADG in pigs gently handled from 11 to 22 weeks of age. However, other studies have reported inconsistent results. For example, Day et al. [71] observed an increase in daily feed intake without changes in ADG or FC during the initial five weeks of gentle handling in growing pigs. Similarly, Wang et al. [72] did not report differences in ADG, feed intake, or FC between gently handled and control pigs from 8 weeks of age until slaughter. These discrepancies highlight that the effects of gentle handling on productive performance are not uniform across time.

Previous research has suggested that long-term gentle handling may have limited effects on pig performance [71, 72]. However, evidence from other species has reported that extended periods of positive human interaction led to improved growth outcomes. For example, a 90-day gentle handling protocol applied during feeding resulted in increased body weight in goats [73], supporting the hypothesis that sustained, consistent positive handling may enhance both welfare and productivity [74].

It is also important to consider that positive human-animal relationships may primarily influence behavioral and physiological factors [2, 23] that are not always directly reflected in conventional productive metrics [72]. Therefore, productive outcomes should be interpreted with caution and should not be used in isolation to assess the impact of human handling [71].

### Composition and diversity of the gut microbiota in pigs

The phyla *Bacteroidota* and *Bacillota* consistently predominated the GM of pigs across all treatments and time points, accounting for over 90% of the total bacterial relative abundance. This pattern is consistent with previous studies in growing pigs [75–77]. At the genus level, *Clostridium sensu stricto 1* was overrepresented across all handling groups at T0, T1, and T2. This result aligns with Xu et al. [78], who identified an enterotype dominated by *Clostridium sensu stricto 1* in Jinhua pigs at 105 days of age. Similarly, Maltecca et al. [79] reported this genus as the most abundant in both Duroc and crossbreed pigs aged 116 to 118 days.

However, the relative abundance of *Clostridium sensu stricto 1* across treatments and time points was highly variable in our study, ranging from 11 to 54%. One plausible explanation for this variability could be related to the gut maturation in growing pigs over time, where *Clostridium sensu stricto 1* has been described as a dominant genus in the GM during the weaning transition phase in commercial piglets [80].

In other studies, *Clostridium sensu stricto 1* has been associated with adverse health conditions in pigs. For instance, higher proportions of this genus have been correlated with poorer fecal consistency [77], as well as detected in pigs affected by necrotizing enterocolitis compared to healthy counterparts [81], suggesting its potential as a microbial biomarker for diarrhea susceptibility. However, it is worth noting that no clinical signs of diarrhea were observed in the animals included in this study. This suggests that variations in *Clostridium sensu stricto 1* abundance may occur independently of overt gastrointestinal signs, and should be interpreted in consideration of the broader microbial community dynamics and host-related factors.

Under natural conditions, GM diversity tends to increase with animal age as the microbial ecosystem matures [82]. However, pigs raised in indoor environments under strict hygienic protocols and subjected to stressful-inducing procedures, such as castration, weaning, or transport, often exhibit reduced microbial diversity [83, 84]. This pattern may explain the decline in GM richness and diversity observed across all groups in our study from T0 to T1, coinciding with weaning and environmental transitions. Interestingly, microbial diversity recovery between T1 and T2 was observed only in the PHH group, whereas both the NHH and CG groups maintained reduced diversity during this period. These results suggest that positive human handling may exert a restorative effect on the GM, counteracting not only the negative impact of repeated aversive interactions but also the consequences of limited human contact, as seen in the CG group.

When comparing treatments, the most pronounced differences in GM were observed at the end of the experimental period (T2), with the NHH group displaying significantly lower GM richness and diversity compared to the PHH group. This supports the notion that repeated exposures to negative handling can disrupt microbial diversity, consistent with previous reports on stress-induced microbiota alterations in other animal species such as birds and fish [12, 85]. Although the absence of a pronounced endocrine response in the NHH group might suggest limited activation of classical stress pathways, the significant microbial changes point toward a biological impact of negative handling. These effects could be mediated through alternative mechanisms, such as immune modulation or enteric nervous system signaling, which influence the gut environment independently of the systemic cortisol output.

In contrast, the PHH group presented a progressive recovery of GM richness and diversity over time, suggesting that positive human interaction may contribute to gut microbial resilience. Changes in bacterial abundance and overall community composition at T2 further support the idea that the quality of human-animal interactions can shape gut microbial dynamics, potentially by mitigating stress-related perturbations or promoting beneficial physiological states. These results are in line with previous studies reporting compositional shifts in the GM of stressed versus non-stressed animals [86–88]. For instance, tail-biting and social stress (e.g., weaning, mixing) have been linked to imbalance in GM composition with implications in health and behavior [14, 86, 87, 89, 90].

Regarding the DA analysis, *Blautia*, *Subdoligranulum*, and *Megasphaera*, were found to be more abundant in the PHH group compared to the NHH group at T2. *Blautia* spp. are known for their numerous beneficial effects on the hosts, including improved digestion and energy intake [91, 92], as well as the production of secondary metabolites that enhance intestinal health and competition with pathogens [93, 94]. Regarding *Subdoligranulum*, its relative abundance has been positively associated with productive performance, particularly higher average daily gain and daily feed intake, and negatively associated with diarrhea incidence in pigs [95]. Although direct correlations between these specific taxa and performance parameters were not established in our study, the presence of these beneficial genera coincided with periods during which we observed significantly higher final BW and ADG in the PHH group. This temporal correspondence suggests a potential link between microbial composition and productive outcomes.

Regarding *Megasphaera*, this genus has been linked to gut health benefits, including recovery from gut-mucosal atrophy in weaned piglets and the prevention of intestinal colonization by antimicrobial-resistant bacterial strains [96, 97]. Its depletion has been observed in contexts of stress and dysbiosis in pigs [87].

In contrast, *Terrisporobacter* was differentially abundant in the NHH group at T2. Consistent with our results, Nguyen et al. [87] reported an enrichment of *Terrisporobacter* in pigs subjected to chronic social stress (i.e., weekly regrouping, reduced space allowance, and limited feeder access), along with a significant depletion of beneficial genera such as *Megasphaera* and *Prevotella* in the stressed animals. These results reinforce the idea that exposure to social stressors induces substantial alterations in the GM, reducing health-promoting taxa while promoting potentially harmful or stress-responsive genera.

Although the association between stress and GM disruption is increasingly supported by animal studies, the influence of positive human-animal interactions on gut microbial profiles remains poorly understood. To date, limited evidence is available regarding the effects of gentle handling on GM of animals. The only study on this topic was conducted by Zhu et al. [98], who evaluated rats challenged with an allergen to induce airway inflammation and subsequently subjected them to daily stroking for 21 days. This intervention significantly increased GM richness and diversity and attenuated allergic airway inflammation compared to non-stroked rats. In addition, stroking increased the relative abundance of *Prevotella*, a commensal genus involved in nutrient absorption and immune function in farm animals, including pigs [99]. Consistent with these results, we observed a significant increase in the abundance of *Prevotella_9* in the PHH group compared to the NHH group. *Prevotella* is a dominant genus in the porcine intestine, where it plays a key role in degrading hemicelluloses and pectin into acetate, an essential short-chain fatty acid for both the host and other gut microbes [87]. However, this increase was detected in only one of the four differential abundance tests performed. It is important to acknowledge that different analytical approaches for differential abundance testing may yield varying results, and the increase in *Prevotella_9*, even if detected in a single test, may indicate a beneficial shift in gut microbial composition associated with positive HAR. Future research should aim to standardize detection approaches and expand current knowledge on the microbiota-mediated effects of gentle handling in livestock species.

The bacterial co-occurrence network analysis revealed notable distinctions in the GM community structures depending on the quality of human handling at T2. The NHH group exhibited the lowest network complexity, with fewer nodes and edges, yet a strikingly high proportion of positive associations (98.01%), suggesting a less interconnected microbial network [100] compared to CG and PHH groups. The bacterial network of this group was dominated by positive interactions between *Clostridium sensu stricto 1* and *Terrisporobacter*, two genera that have been previously associated with intestinal disorders and stress-related dysbiosis [77, 87]. These results suggest that repeated negative human handling may favor the expansion of potentially harmful taxa while suppressing overall microbial heterogeneity and stability.

Conversely, more complex and diverse networks were observed in the PHH and CG groups, with higher numbers of nodes, lower positive edge proportions, and associations involving health-promoting genera such as *Lactobacillus.* This genus was unconnected in the NHH network, potentially due to antagonistic interactions with *Clostridium sensu stricto 1*, as evidenced by their negative correlation in the CG and PHH groups. The lack of *Lactobacillus* connectivity in the NHH group could reflect a disrupted microbial balance and a loss of probiotic function, which is critical for gut barrier integrity, immune modulation, and competitive exclusion of pathogens [101–104]. In contrast, the presence and connectivity of *Lactobacillus* in the PHH and CG groups may indicate greater microbial resilience and contribute to the observed improvements in welfare and productive outcomes [105, 106].

Finally, based on these findings, future studies should consider exploring additional microbial niches, such as the nasal microbiota [107, 108], which may also be affected by the quality of human handling. Expanding the analysis beyond the gut could offer a more comprehensive understanding of how management practices shape microbial dynamics across different body systems and developmental stages.

## Conclusions

This study provides evidence that the quality of human–animal relationship has a measurable impact on the GM, stress response, and productive parameters in pigs. Positive handling was associated with reduced hair cortisol concentrations, improved performance outcomes, and increased microbial diversity and network complexity, suggesting a protective effect against stress. In contrast, negative handling did not elicit measurable increases in hair cortisol concentrations but was linked to lower microbial diversity, reduced richness, and less interconnected bacterial communities. Specific taxa such as *Blautia, Megasphaera*, *Subdoligranulum*, and *Terrisporobacter* emerged as potential microbial biomarkers reflecting welfare states under contrasting human handling conditions. These findings reinforce the role of the microbiota–gut–brain axis in animal welfare and indicate that promoting positive human–animal relationships may modulate GM and improve both welfare and productivity in intensive farming systems.

## Supplementary Information

Below is the link to the electronic supplementary material.

Supplementary Material 1

Supplementary Material 2

Supplementary Material 3

Supplementary Material 4

Supplementary Material 5

Supplementary Material 6

Supplementary Material 7

Supplementary Material 8

Supplementary Material 9

Supplementary Material 10

Supplementary Material 11

Supplementary Material 12

Supplementary Material 13

## Data Availability

The dataset used in this study was deposited and available in the Sequence Read Archive (SRA) repository of the National Center for Biotechnology Information (NCBI) under the BioProject accession number PRJNA1161000. Other data in this study are available from the authors upon reasonable request.

## Abbreviations

HAR: Human Animal relationship
GM: Gut microbiota
PHH: Positive human handling
NHH: Negative human handling
CG: Control Group
T0: day 16
T1: day 37
T2: day 65
DA: Differential abundance
PCoA: Principal coordinate analysis (PCoA)
