# Supplementary material for: The role of human–pig interactions in modulating gut microbiota, stress, and performance
Source: Porcine Health Manag. 2025 Oct 23;11:51. doi: 10.1186/s40813-025-00465-2 (PMC12548226; doi:10.1186/s40813-025-00465-2)
Supplement: Supplementary file 1 — Supplementary Material 1 [file 40813_2025_465_MOESM1_ESM.docx]

**Additional file 1**. **Composition of the diet provided to the animals during the study.**

| Composition | Use |
| --- | --- |
| Maize (grains) (%) | 58.214 |
| Soybean meal 47% | 28.155 |
| Rapeseed meal (%) | 8.000 |
| Vegetable oil (olein) (%) | 2.080 |
| Molasses (%) | 1.427 |
| Calcium phosphate 18 (%) | 0.865 |
| Salt (%) | 0.600 |
| Ground seashell (%) | 0.352 |
| Nucleo PLIN 110 (%) | 0.303 |
| Linoleic acid (%) | 2.871 |
| Crude fat (%) | 4.350 |
| NFE (%) | 53.292 |
| Starch (%) | 37.925 |
| Sugars (%) | 5.161 |
| Metabolizable energy (Kcal/kg) | 3379.809 |
| Net energy of pigs (Kcal/kg) | 2273.541 |
| Lys Dig. Pigs/ME | 3.071 |
| Lys Dig. Pigs/NE | 4.710 |
| Crude fiber (%) | 3.857 |
| NDF (%) | 14.977 |
| ADF (%) | 6.533 |
| ADL (%) | 1.726 |
| Humidity (%) | 12.094 |
| Total minerals (%) | 4.987 |
| Calcium (%) | 0.700 |
| Total phosphorus (%) | 0.583 |
| Available phosphorus (%) | 0.504 |
| Magnesium (%) | 0.190 |
| Sodium (%) | 0.295 |
| Chlorine (%) | 0.431 |
| Potassium (%) | 0.951 |
| Iron (mg/kg) | 295.439 |
| Copper (mg/kg) | 18.761 |
| Manganese (mg/kg) | 39.239 |
| Selenium (mg/kg) | 0.299 |
| Iodine (mg/kg) | 3.354 |
| Zinc (mg/kg) | 184.409 |
| Electrolyte balance (mEq/kg) | 250.000 |
| Crude protein (%) | 21.818 |
| Digestible protein (%) | 17.779 |
| Lys (%) | 1.159 |
| Lys Dig. (%) | 1.014 |
| Thr Dig. (%) | 0.725 |
| Met Dig. (%) | 0.315 |
| Met+Cys Dig. (%) | 0.646 |
| Trp Dig. (%) | 0.237 |
| Ile Dig. (%) | 0.798 |
| Val Dig. (%) | 0.898 |
| Leu Dig. (%) | 1.577 |
| Phe Dig. (%) | 1.565 |
| Phe+Tyr Dig. (%) | 1.565 |
| His Dig. (%) | 0.507 |
| Arg Dig. (%) | 1.305 |
| Biotin (mg/kg) | 0.204 |
| Choline (mg/kg) | 2309.698 |
| Folic acid (mg/kg) | 0.469 |
| Niacin (mg/kg) | 71.488 |
| Pantothenic acid (mg/kg) | 38.099 |
| Riboflavin (mg/kg) | 10.988 |
| Thiamine (mg/kg) | 3.240 |
| Vit. B6 (mg/kg) | 5.695 |
| Vit. B12 (mg/kg) | 0.151 |
| Vit. E (UI/kg) | 35.269 |
| Vit. A (UI/kg) | 7000.000 |
| Vit. D (UI/kg) | 1215.000 |
| Vit. K (mg/kg) | 1.716 |
| MIX (%) | 100.000 |
| Total Calcium/Phosphorus | 1.200 |
| Thr Dig./Lys Dig. | 0.715 |
| Met+CysDig./Lys Dig. | 0.637 |
| Trp Dig./Lys Dig. | 0.234 |
| Ile Dig./Lys Dig. | 0.787 |
| Val Dig./Lys Dig. | 0.885 |
| Leu Dig./Lys Dig. | 1.555 |
| Phe+Tyr Dig./Lys Dig. | 1.543 |
| His Dig./Lys Dig. | 0.500 |

%: percentage; Kcal/kg: kilocalories per kilogram; mg/kg: milligrams per kilogram; mEq/kg: milliequivalents per kilogram; UI/kg: International Units per kilogram; NFE: Nitrogen-free extract; ME: Metabolizable energy; NE: Net energy; NDF: Neutral detergent fiber; ADF: Acid detergent fiber; ADL: Acid detergent lignin; Arg: Arginine; Trp: Tryptophan; Thr: Threonine; Lys: Lysine; Met: Methionine; Cys: Cysteine; Ile: Isoleucine; Val: Valine; Leu: Leucine; Phe: Phenylalanine; Tyr: Tyrosine; His: Histidine; Dig: Digestible.
