# Supplementary material for: The role of human–pig interactions in modulating gut microbiota, stress, and performance
Source: Porcine Health Manag. 2025 Oct 23;11:51. doi: 10.1186/s40813-025-00465-2 (PMC12548226; doi:10.1186/s40813-025-00465-2)
Supplement: Supplementary file 2 — Supplementary Material 2 [file 40813_2025_465_MOESM2_ESM.docx]

**Additional file 2**. **Schematic representation of the processing of cortisol samples from pig hair.**

| 1.- Hair weight (250 mg) measured using a digital scale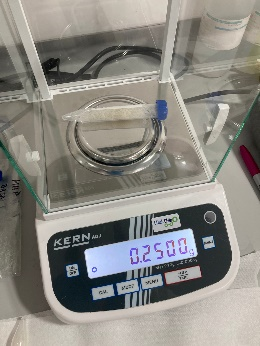 | 2.- Hair washed three times with isopropanol and vortexed at 3000 rpm for 2.5 min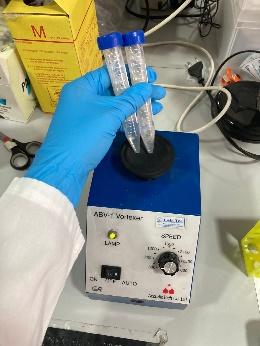 | 3.- Hair samples dried at room temperature in a vertical laminar flow hood for 5 days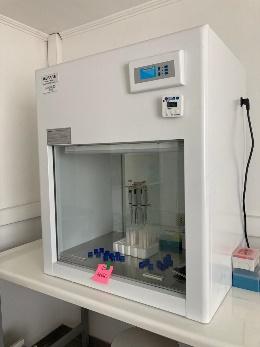 | 4.- Samples stored at -80º C until cortisol extraction processing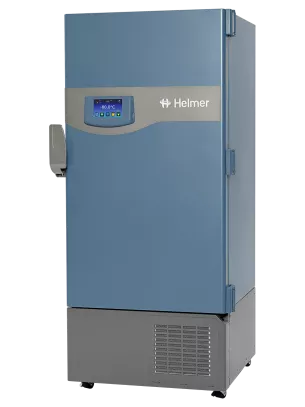 | 5.- Hair finely cut with a scalpel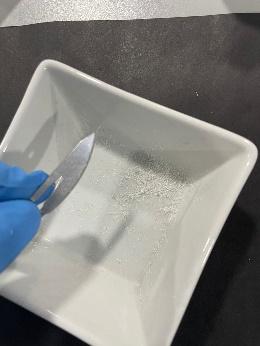 |
| --- | --- | --- | --- | --- |
| 6.- 40 mg of powdered hair weighed and carefully placed into a 2-ml microcentrifuge tube with four 2 mm zirconia balls 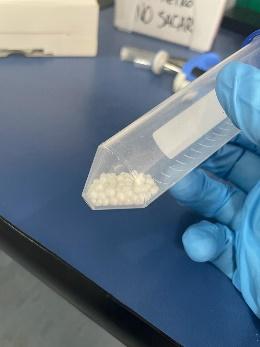 | 7.- Hair pulverized into a ball mill at 2 for 15 min, 30 Hz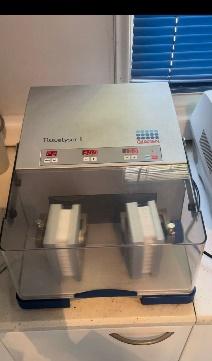 | 8.- Pulverized hair mixed with 1 mL of methanol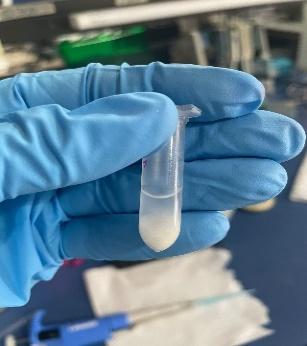 | 9.- Samples incubated at 100 rpm, 36°C for 24 h with slow rotation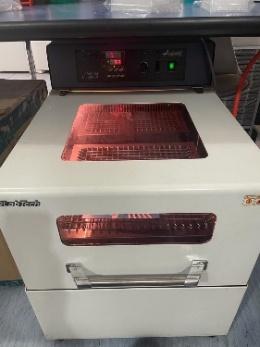 | 10.- Samples centrifugated at 9500 rpm for 5 min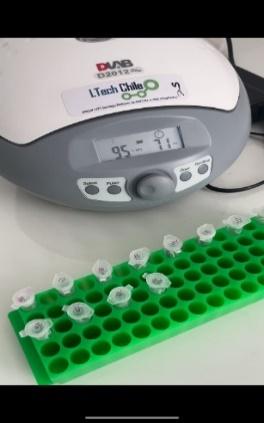 |
| 11.- Supernatant transferred into and Eppendorf tube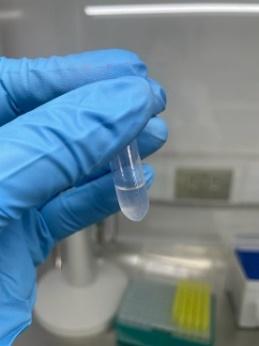 | 12.- Hair samples dried at room temperature in a vertical laminar flow hood for 24 h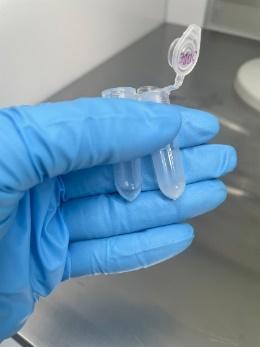 | 13. Reconstitution of the samples in 0.250 ml of PBS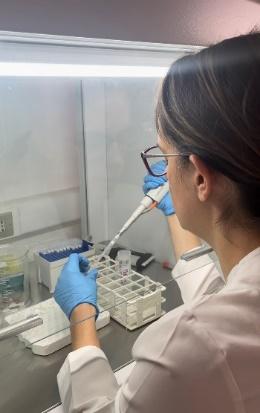 | 14.- Samples vortexed at 1200 rpm for 30 s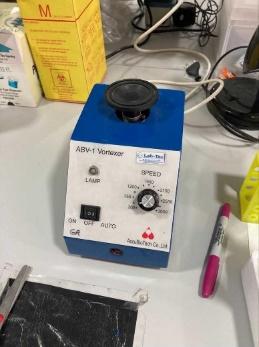 | 15.- Samples stored at -20°C until analysis with the Cortisol ELISA kit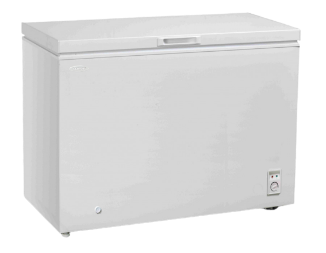 |
| 16.- Samples processed in duplicated using the ELISA Kit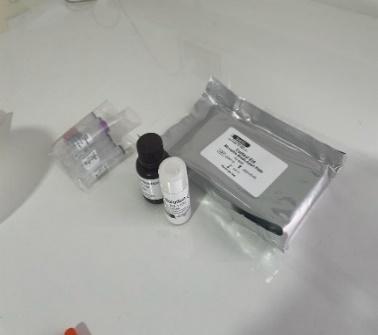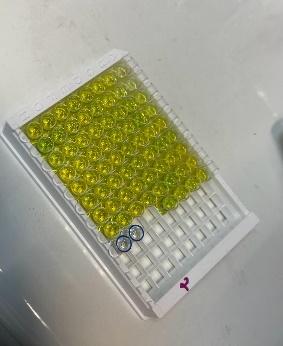 | | 17.- Optical density read by a microplate reader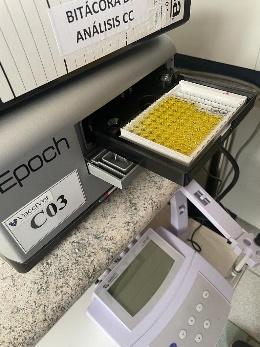 | 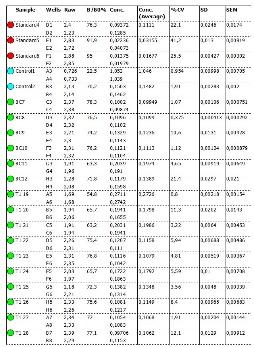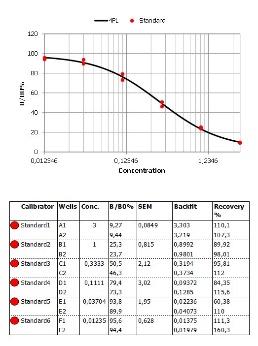  18. Analysis of the Data obtained from the microplate reader | |
