# Supplementary material for: The role of human–pig interactions in modulating gut microbiota, stress, and performance
Source: Porcine Health Manag. 2025 Oct 23;11:51. doi: 10.1186/s40813-025-00465-2 (PMC12548226; doi:10.1186/s40813-025-00465-2)
Supplement: Supplementary file 3 — Supplementary Material 3 [file 40813_2025_465_MOESM3_ESM.docx]

**Additional file 3**. **Phyla and top ten genera in each treatment group (CG, PHH, and NHH) at T0, T1 and T2.** Each genus is ranked from highest to lowest abundance in the control group (CG), positive human handling (PHH) and negative human handling (NHH) groups. Results are expressed as mean (%) and standard deviation (SD).

|  |  | CG | | PHH | | | NHH | |
| --- | --- | --- | --- | --- | --- | --- | --- | --- |
|  | Phylum | Mean | SD | Mean | SD | Mean | | SD |
| T0 | Bacillota | 82.315 | 6.351 | 86.481 | 6.647 | 79.963 | | 6.602 |
|  | Bacteroidota | 17.457 | 6.333 | 13.340 | 6.691 | 19.874 | | 6.621 |
|  | Proteobacteria | 0.144 | 0.134 | 0.086 | 0.074 | 0.093 | | 0.061 |
|  | Patescibacteria | 0.077 | 0.066 | 0.067 | 0.085 | 0.060 | | 0.077 |
|  | Euryarchaeota | 0.007 | 0.0179 | 0.026 | 0.041 | 0.010 | | 0.022 |
| T1 | Bacillota | 91.456 | 4.456 | 91.497 | 3.614 | 93.312 | | 3.848 |
|  | Bacteroidota | 8.269 | 4.530 | 8.362 | 3.598 | 6.496 | | 3.919 |
|  | Proteobacteria | 0.139 | 0.099 | 0.074 | 0.069 | 0.077 | | 0.075 |
|  | Patescibacteria | 0.072 | 0.059 | 0.046 | 0.054 | 0.065 | | 0.048 |
|  | Euryarchaeota | 0.065 | 0.074 | 0.022 | 0.044 | 0.050 | | 0.096 |
| T2 | Bacillota | 93.604 | 2.604 | 90.946 | 3.249 | 93.971 | | 3.329 |
|  | Bacteroidota | 6.218 | 2.592 | 8.975 | 3.289 | 5.933 | | 3.347 |
|  | Patescibacteria | 0.089 | 0.101 | 0.048 | 0.046 | 0.060 | | 0.065 |
|  | Proteobacteria | 0.069 | 0.082 | 0.022 | 0.035 | 0.022 | | 0.043 |
|  | Euryarchaeota | 0.019 | 0.028 | 0.010 | 0.033 | 0.014 | | 0.029 |

|  |  | CG | | PHH | | | NHH | |
| --- | --- | --- | --- | --- | --- | --- | --- | --- |
|  | Genus | Mean | SD | Mean | SD | Mean | | SD |
| T0 | *Clostridium sensu stricto 1* | 20.980 | 19.021 | 16.410 | 17.223 | 11.322 | | 12.345 |
|  | *Lactobacillus* | 17.099 | 16.556 | 19.193 | 12.613 | 18.778 | | 10.078 |
|  | *Prevotella_9* | 12.998 | 7.346 | 8.653 | 6.530 | 16.153 | | 6.673 |
|  | *Terrisporobacter* | 6.688 | 6.380 | 5.401 | 4.161 | 5.342 | | 2.268 |
|  | *HT002* | 4.670 | 4.836 | 8.997 | 9.206 | 4.781 | | 3.269 |
|  | *Blautia* | 4.133 | 3.016 | 3.569 | 2.761 | 6.355 | | 3.790 |
|  | *Subdoligranulum* | 2.902 | 1.800 | 2.587 | 3.158 | 2.879 | | 1.920 |
|  | *Phascolarctobacterium* | 2.717 | 0.823 | 2.454 | 9.736 | 2.281 | | 0.638 |
|  | *Coprococcus* | 2.393 | 1.125 | 2.673 | 1.401 | 2.589 | | 0.951 |
|  | *Agathobacter* | 2.129 | 2.775 | 1.441 | 1.658 | 1.831 | | 2.114 |
| T1 | *Clostridium sensu stricto 1* | 48.106 | 14.607 | 51.422 | 13.480 | 49.506 | | 14.780 |
|  | *Lactobacillus* | 10.347 | 8.196 | 7.426 | 7.539 | 7.476 | | 7.128 |
|  | *Terrisporobacter* | 8.850 | 2.941 | 9.965 | 2.840 | 8.813 | | 2.853 |
|  | *Prevotella_9* | 4.875 | 4.215 | 5.525 | 3.813 | 3.789 | | 3.636 |
|  | *HT002* | 2.778 | 3.041 | 3.224 | 4.733 | 1.474 | | 1.328 |
|  | *Coprococcus* | 2.262 | 0.928 | 2.025 | 0.771 | 2.029 | | 0.643 |
|  | *Shuttleworthia* | 1.715 | 2.093 | 0.983 | 1.239 | 1.431 | | 3.499 |
|  | *UCG-005* | 1.572 | 0.796 | 1.130 | 0.739 | 1.127 | | 0.616 |
|  | *Agathobacter* | 1.514 | 1.555 | 0.850 | 0.811 | 0.622 | | 0.710 |
|  | *Blautia* | 1.460 | 0.938 | 1.745 | 1.976 | 1.472 | | 1.419 |
| T2 | *Clostridium sensu stricto 1* | 43.076 | 18.022 | 36.806 | 14.426 | 53.959 | | 13.787 |
|  | *Lactobacillus* | 10.803 | 8.324 | 10.272 | 11.371 | 5.506 | | 5.861 |
|  | *HT002* | 9.194 | 6.565 | 6.221 | 6.280 | 2.772 | | 3.317 |
|  | *Terrisporobacter* | 7.700 | 3.271 | 6.539 | 2.713 | 10.791 | | 3.757 |
|  | *Prevotella_9* | 3.282 | 2.580 | 6.196 | 3.332 | 3.251 | | 3.134 |
|  | *Coprococcus* | 2.146 | 1.014 | 1.991 | 0.714 | 1.499 | | 0.516 |
|  | *Megasphaera* | 2.129 | 2.742 | 4.005 | 2.708 | 1.023 | | 1.758 |
|  | *Blautia* | 1.992 | 1.573 | 4.000 | 1.772 | 1.570 | | 1.424 |
|  | *[Ruminococcus] gauvreauii group* | 1.664 | 1.158 | 1.619 | 0.388 | 1.224 | | 0.583 |
|  | *Subdoligranulum* | 1.630 | 0.974 | 2.291 | 1.232 | 1.028 | | 0.997 |
