# Supplementary material for: The role of human–pig interactions in modulating gut microbiota, stress, and performance
Source: Porcine Health Manag. 2025 Oct 23;11:51. doi: 10.1186/s40813-025-00465-2 (PMC12548226; doi:10.1186/s40813-025-00465-2)
Supplement: Supplementary file 4 — Supplementary Material 4 [file 40813_2025_465_MOESM4_ESM.docx]

**Additional file 4**. **Taxonomic composition of bacterial communities at genera level present in fecal samples from pigs subjected to different human handling over time.** Different colors represent distinct genera. CG = control group; NNH = negative human handling; PHH = positive human handling; T0 = day 16 (baseline); T1 = day 37; T2 = day 65.

**
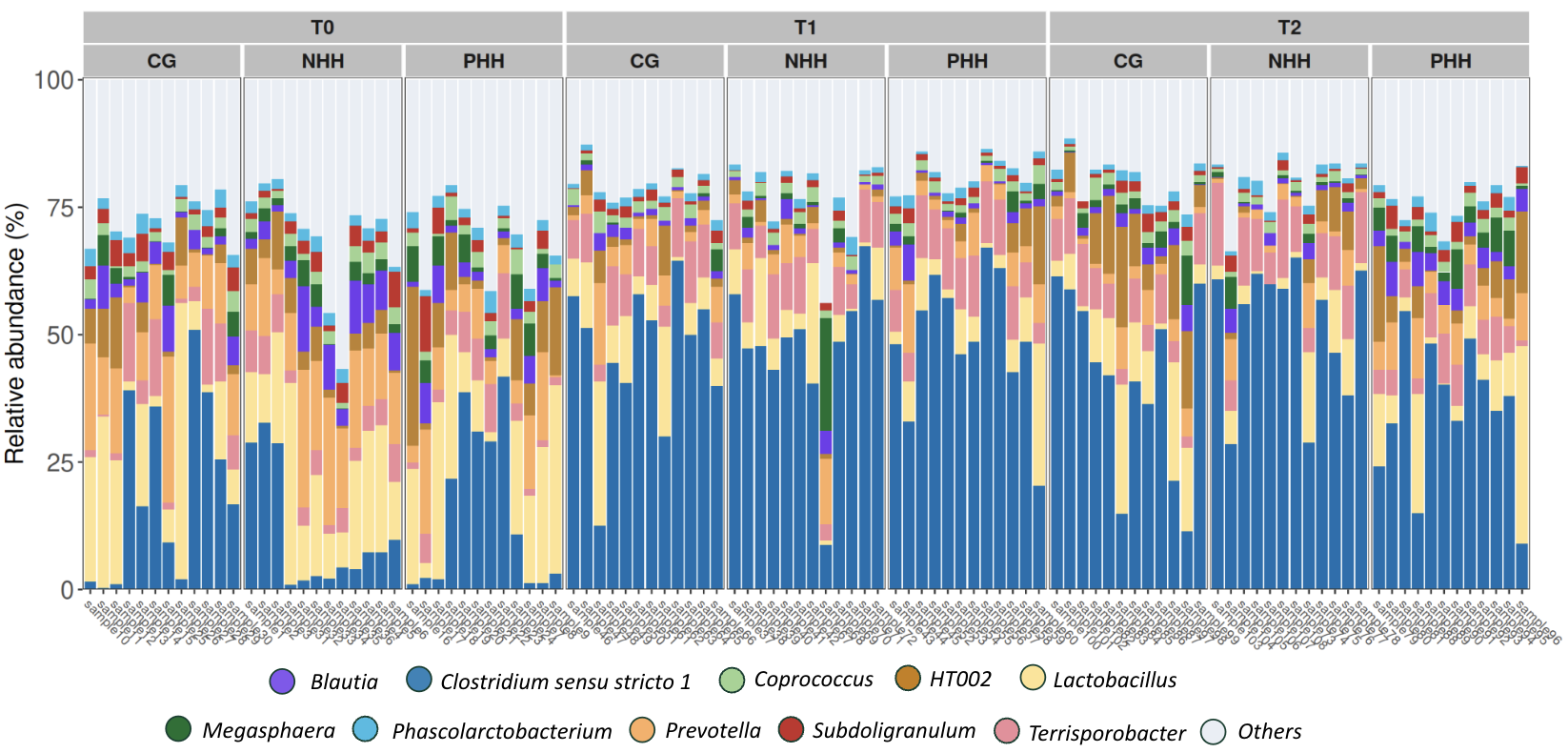
**
