# Supplementary material for: The role of human–pig interactions in modulating gut microbiota, stress, and performance
Source: Porcine Health Manag. 2025 Oct 23;11:51. doi: 10.1186/s40813-025-00465-2 (PMC12548226; doi:10.1186/s40813-025-00465-2)
Supplement: Supplementary file 5 — Supplementary Material 5 [file 40813_2025_465_MOESM5_ESM.docx]

**Additional file 5**. **Effects of explanatory variables using the ‘Anova’ function (‘car’ R package), running Type II Wald chi-square trials.** All linear models were computed using the ‘lmer’ function, considering repeated observations as random factors (individual). All the models by alpha-diversity metrics (dependent variable) and the explanatory variables time and treatment and the interaction between time and treatment are shown. *P*-*values* below 0.05 were considered significant.

| Predictor | Chis | Df | Pr.Chisq. |
| --- | --- | --- | --- |
| **Shannon** |  |  |  |
| Time | 102.21 | 2 | **<0.0001** |
| Treatment | 1.53 | 2 | 0.46 |
| Time*Treatment | 23.20 | 4 | **0.0001** |
| **Simpson** |  |  |  |
| Time | 77.73 | 2 | **<0.0001** |
| Treatment | 1.96 | 2 | 0.37 |
| Time*Treatment | 19.58 | 4 | **0.0006** |
| **Pielou** |  |  |  |
| Time | 82.11 | 2 | **<0.0001** |
| Treatment | 1.10 | 2 | 0.57 |
| Time*Treatment | 20.17 | 4 | **0.0004** |
| **Observed Species** |  |  |  |
| Time | 103.76 | 2 | **<0.0001** |
| Treatment | 1.35 | 2 | 0.50 |
| Time*Treatment | 20.55 | 4 | **0.0003** |
