# Supplementary material for: The role of human–pig interactions in modulating gut microbiota, stress, and performance
Source: Porcine Health Manag. 2025 Oct 23;11:51. doi: 10.1186/s40813-025-00465-2 (PMC12548226; doi:10.1186/s40813-025-00465-2)
Supplement: Supplementary file 6 — Supplementary Material 6 [file 40813_2025_465_MOESM6_ESM.docx]

**Additional file 6. Post hoc tests displaying significant interactions of explanatory variables (time*treatment) on alpha diversity metrics.** The table presents both within- and between-treatment effects for each alpha-diversity metric (Shannon, Simpson, Pielou, and Observed species indices). Post hoc tests were computed using the 'lsmeans' function and Tukey correction for multiple comparisons.

|  |  | estimate | SE | df | t.ratio | *P*- value |
| --- | --- | --- | --- | --- | --- | --- |
| **Shannon** |  |  |  |  |  |  |
| **Time** | **Treatment** |  |  |  |  |  |
| T0 - T1 | CG | 0.43 | 0.10 | 66 | 4.16 | **0.0002** |
| T0 - T2 | CG | 0.32 | 0.10 | 66 | 3.07 | **0.008** |
| T1 - T2 | CG | -0.11 | 0.10 | 66 | -1.09 | 0.52 |
| T0 - T1 | NHH | 0.76 | 0.10 | 66 | 7.29 | **<0.0001** |
| T0 - T2 | NHH | 0.82 | 0.10 | 66 | 7.94 | **<0.0001** |
| T1 - T2 | NHH | 0.06 | 0.10 | 66 | 0.64 | 0.79 |
| T0 - T1 | PHH | 0.55 | 0.10 | 66 | 5.28 | **<0.0001** |
| T0 - T2 | PHH | 0.18 | 0.10 | 66 | 1.78 | 0.18 |
| T1 - T2 | PHH | -0.36 | 0.10 | 66 | -3.50 | **0.002** |
| **Treatment** | **Time** |  |  |  |  |  |
| CG - NHH | T0 | -0.23 | 0.11 | 33.19 | -1.98 | 0.13 |
| CG - PHH | T0 | -0.05 | 0.11 | 33.19 | -0.44 | 0.89 |
| NHH - PHH | T0 | 0.18 | 0.11 | 33.19 | 1.53 | 0.28 |
| CG - NHH | T1 | 0.09 | 0.11 | 33.19 | 0.77 | 0.72 |
| CG - PHH | T1 | 0.06 | 0.11 | 33.19 | 0.53 | 0.85 |
| NHH - PHH | T1 | -0.02 | 0.11 | 33.19 | -0.23 | 0.97 |
| CG - NHH | T2 | 0.27 | 0.11 | 33.19 | 2.29 | 0.06 |
| CG - PHH | T2 | -0.18 | 0.11 | 33.19 | -1.57 | 0.26 |
| NHH - PHH | T2 | -0.45 | 0.11 | 33.19 | -3.87 | **0.001** |
| **Simpson** |  |  |  |  |  |  |
| Time | Treatment |  |  |  |  |  |
| T0 - T1 | CG | 0.001 | 0.0005 | 66 | 3.67 | **0.001** |
| T0 - T2 | CG | 0.001 | 0.0005 | 66 | 2.31 | 0.06 |
| T1 - T2 | CG | -0.0006 | 0.0005 | 66 | -1.36 | 0.36 |
| T0 - T1 | NHH | 0.003 | 0.0005 | 66 | 6.13 | **<0.0001** |
| T0 - T2 | NHH | 0.003 | 0.0005 | 66 | 6.88 | **<0.0001** |
| T1 - T2 | NHH | 0.0003 | 0.0005 | 66 | 0.74 | 0.73 |
| T0 - T1 | PHH | 0.002 | 0.0005 | 66 | 4.99 | **<0.0001** |
| T0 - T2 | PHH | 0.0007 | 0.0005 | 66 | 1.43 | 0.32 |
| T1 - T2 | PHH | -0.001 | 0.0005 | 66 | -3.55 | **0.002** |
| **Treatment** | **Time** |  |  |  |  |  |
| CG - NHH | T0 | -0.0008 | 0.0005 | 32.92 | -1.52 | 0.29 |
| CG - PHH | T0 | -0.0003 | 0.0005 | 32.92 | -0.58 | 0.83 |
| NHH - PHH | T0 | 0.0005 | 0.0005 | 32.92 | 0.94 | 0.61 |
| CG - NHH | T1 | 0.0003 | 0.0005 | 32.92 | 0.62 | 0.80 |
| CG - PHH | T1 | 0.0003 | 0.0005 | 32.92 | 0.57 | 0.83 |
| NHH - PHH | T1 | 0.00005 | 0.0005 | 32.92 | -0.05 | 0.99 |
| CG - NHH | T2 | 0.001 | 0.0005 | 32.92 | 2.47 | **0.04** |
| CG - PHH | T2 | -0.0007 | 0.0005 | 32.92 | -1.35 | 0.37 |
| NHH - PHH | T2 | -0.002 | 0.0005 | 32.92 | -3.82 | **0.001** |
| **Pielou** |  |  |  |  |  |  |
| **Time** | **Treatment** |  |  |  |  |  |
| T0 - T1 | CG | 0.03 | 0.009 | 66 | 3.79 | **0.0009** |
| T0 - T2 | CG | 0.01 | 0.009 | 66 | 1.91 | 0.14 |
| T1 - T2 | CG | -0.01 | 0.009 | 66 | -1.88 | 0.15 |
| T0 - T1 | NHH | 0.06 | 0.009 | 66 | 6.56 | **< 0.0001** |
| T0 - T2 | NHH | 0.06 | 0.009 | 66 | 6.89 | **< 0.0001** |
| T1 - T2 | NHH | 0.003 | 0.009 | 66 | 0.32 | 0.94 |
| T0 - T1 | PHH | 0.04 | 0.009 | 66 | 5.07 | **< 0.0001** |
| T0 - T2 | PHH | 0.01 | 0.009 | 66 | 1.40 | 0.34 |
| T1 - T2 | PHH | -0.03 | 0.009 | 66 | -3.67 | **0.001** |
| **Treatment** | **Time** |  |  |  |  |  |
| CG - NHH | T0 | -0.02 | 0.01 | 31.14 | -1.87 | 0.16 |
| CG - PHH | T0 | -0.007 | 0.01 | 31.14 | -0.62 | 0.80 |
| NHH - PHH | T0 | 0.01 | 0.01 | 31.14 | 1.25 | 0.43 |
| CG - NHH | T1 | 0.005 | 0.01 | 31.14 | 0.51 | 0.86 |
| CG - PHH | T1 | 0.005 | 0.01 | 31.14 | 0.48 | 0.87 |
| NHH - PHH | T1 | -0.0002 | 0.01 | 31.14 | -0.02 | 0.99 |
| CG - NHH | T2 | 0.02 | 0.01 | 31.14 | 2.41 | 0.06 |
| CG - PHH | T2 | -0.01 | 0.01 | 31.14 | -1.05 | 0.54 |
| NHH - PHH | T2 | -0.03 | 0.01 | 31.14 | -3.47 | **0.004** |
| **Observed Species** |  |  |  |  |  |  |
| **Time** | **Treatment** |  |  |  |  |  |
| T0 - T1 | CG | 210.25 | 52.45 | 66 | 4.00 | **0.0004** |
| T0 - T2 | CG | 223.41 | 52.45 | 66 | 4.25 | **0.0001** |
| T1 - T2 | CG | 13.16 | 52.45 | 66 | 0.25 | 0.96 |
| T0 - T1 | NHH | 380.08 | 52.45 | 66 | 7.24 | **< 0.0001** |
| T0 - T2 | NHH | 426.58 | 52.45 | 66 | 8.13 | **< 0.0001** |
| T1 - T2 | NHH | 46.5 | 52.45 | 66 | 0.88 | 0.65 |
| T0 - T1 | PHH | 245 | 52.45 | 66 | 4.67 | **< 0.0001** |
| T0 - T2 | PHH | 112.75 | 52.45 | 66 | 2.14 | 0.08 |
| T1 - T2 | PHH | -132.25 | 52.45 | 66 | -2.52 | **0.03** |
| **Treatment** | **Time** |  |  |  |  |  |
| CG - NHH | T0 | -114.08 | 56.70 | 39.32 | -2.01 | 0.12 |
| CG - PHH | T0 | -5.91 | 56.70 | 39.32 | -0.10 | 0.99 |
| NHH - PHH | T0 | 108.16 | 56.70 | 39.32 | 1.90 | 0.14 |
| CG - NHH | T1 | 55.75 | 56.70 | 39.32 | 0.98 | 0.59 |
| CG - PHH | T1 | 28.83 | 56.70 | 39.32 | 0.50 | 0.86 |
| NHH - PHH | T1 | -26.91 | 56.70 | 39.32 | -0.47 | 0.88 |
| CG - NHH | T2 | 89.08 | 56.70 | 39.32 | 1.57 | 0.26 |
| CG - PHH | T2 | -116.58 | 56.70 | 39.32 | -2.05 | 0.11 |
| NHH - PHH | T2 | -205.66 | 56.70 | 39.32 | -3.62 | **0.002** |
