# Supplementary material for: The role of human–pig interactions in modulating gut microbiota, stress, and performance
Source: Porcine Health Manag. 2025 Oct 23;11:51. doi: 10.1186/s40813-025-00465-2 (PMC12548226; doi:10.1186/s40813-025-00465-2)
Supplement: Supplementary file 7 — Supplementary Material 7 [file 40813_2025_465_MOESM7_ESM.docx]

**Additional file 7. Mean and standard error of the mean (SEM) of the alpha-diversity metrics.** The table displays each alpha diversity measure (Shannon, Simpson, Pielou, and Observed Species indices) from pigs' fecal samples across different treatments (PHH, NHH, and CG) and sampling periods (T0, T1, and T2).

| Alpha-diversity metrics | | | PHH | | NHH | CG |
| --- | --- | --- | --- | --- | --- | --- |
|  | | | Mean (SEM) | | Mean (SEM) | Mean (SEM) |
| **T0 (Day 16)** | | | | |  |  |
| Shannon | | | 6.57 (0.084) | | 6.75 (0.084) | 6.52 (0.084) |
| Simpson | | | 0.998 (0.000409) | | 0.998 (0.000409) | 0.997 (0.000409) |
| Pielou | | | 0.925 (0.008) | | 0.939 (0.008) | 0.918 (0.008) |
| Observed Species | | | 1220.58 (40.09) | | 1328.75 (40.09) | 1214.66 (40.09) |
| **T1 (Day 37)** | | | | |  |  |
| Shannon | | | 6.02 (0.084) | | 5.99 (0.084) | 6.08 (0.084) |
| Simpson | | | 0.995 (0.000409) | | 0.995 (0.000409) | 0.995 (0.000409) |
| Pielou | | | 0.876 (0.008) | | 0.876 (0.008) | 0.881 (0.008) |
| Observed Species | | | 975.58 (40.09) | | 948.66 (40.09) | 1004.41 (40.09) |
| **T2 (Day 65)** | | | | |  |  |
| Shannon | | 6.38 (0.084) | | | 5.92 (0.084) | 6.20 (0.084) |
| Simpson | | 0.997 (0.000409) | | | 0.995 (0.000409) | 0.996 (0.000409) |
| Pielou | | 0.912 (0.008) | | | 0.872 (0.008) | 0.900 (0.008) |
| Observed Species | 1107.83 (40.09) | | | 902.16 (40.09) | | 991.25 (40.09) |

PHH= Positive human handling; NHH= Negative human handling; CG= Control group.
