# Supplementary material for: The role of human–pig interactions in modulating gut microbiota, stress, and performance
Source: Porcine Health Manag. 2025 Oct 23;11:51. doi: 10.1186/s40813-025-00465-2 (PMC12548226; doi:10.1186/s40813-025-00465-2)
Supplement: Supplementary file 9 — Supplementary Material 9 [file 40813_2025_465_MOESM9_ESM.docx]

**Additional file 9**. **Jaccard dissimilarities of pigs´ fecal samples displayed by principal coordinate analysis (PCoA) at different sampling periods (T0, T1, and T2).** The samples were rarefied to 3479 sequences per sample. CG = control group; NHH = negative human handling; PHH = positive human handling. T0 = day 16 (baseline); T1 = day 37; T2 = day 65.


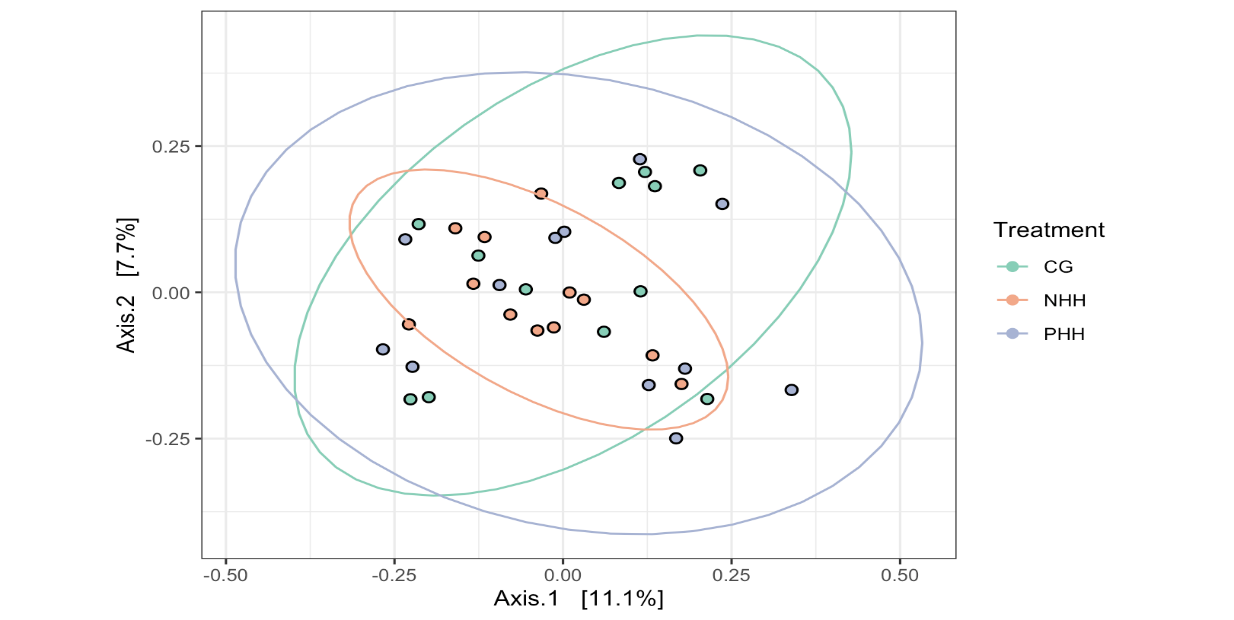


**T0**


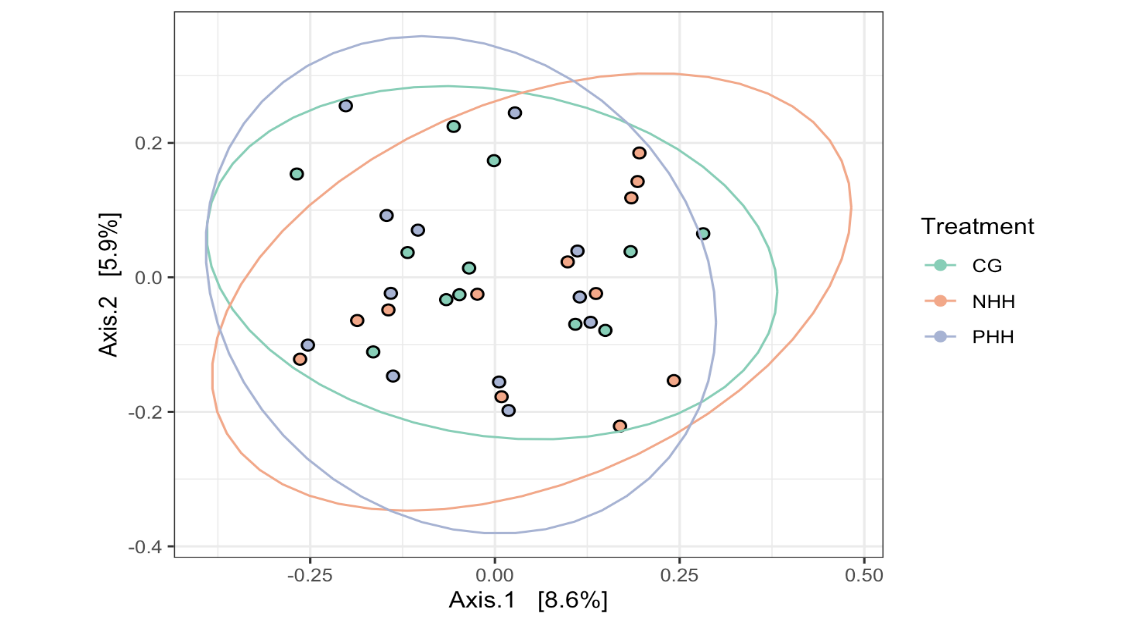


**T1**


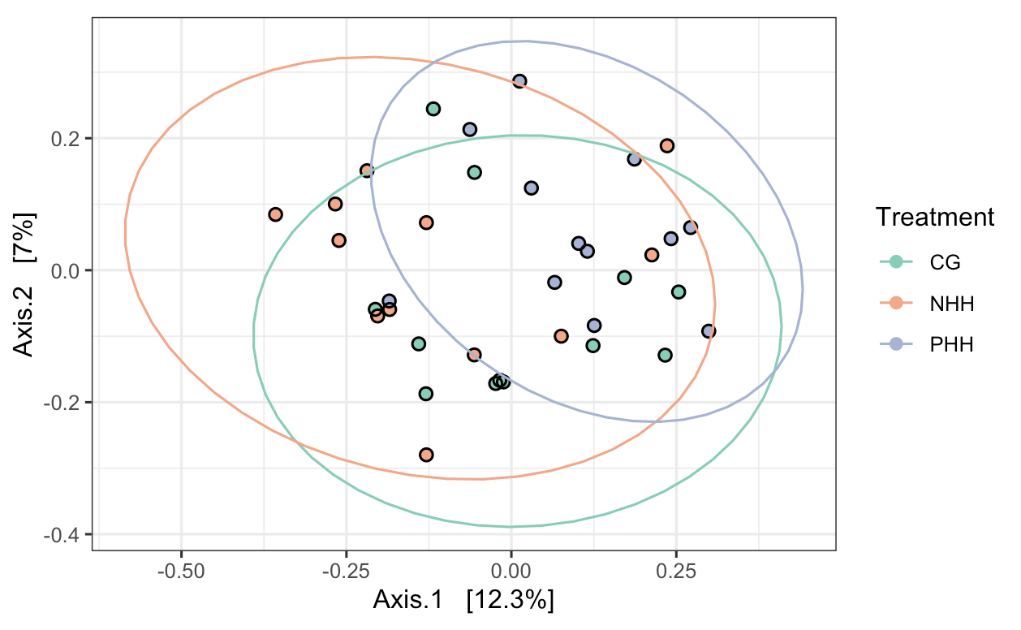


**T2**
