# Supplementary material for: The role of human–pig interactions in modulating gut microbiota, stress, and performance
Source: Porcine Health Manag. 2025 Oct 23;11:51. doi: 10.1186/s40813-025-00465-2 (PMC12548226; doi:10.1186/s40813-025-00465-2)
Supplement: Supplementary file 10 — Supplementary Material 10 [file 40813_2025_465_MOESM10_ESM.docx]

**Additional file 10**. **Scoring table of the four DA tests comparing genera abundances between handling treatments by pairwise comparisons (CG-PHH, CG-NHH, and NHH-PHH) groups at different sampling periods (T0, T1, and T2).** Score reports how many of the four DA tests detected a genus significantly abundant between compared groups.

| **Compared groups** | **CG-PHH** | | | **CG-NHH** | | | **NHH-PHH** | | |
| --- | --- | --- | --- | --- | --- | --- | --- | --- | --- |
| **Time  Genus** | **T0** | **T1** | **T2** | **T0** | **T1** | **T2** | **T0** | **T1** | **T2** |
| *Clostridium sensu stricto 1* |  |  |  |  |  |  |  |  | 1 |
| *Lactobacillus* |  |  |  |  |  |  |  |  |  |
| *Terrisporobacter* |  |  |  |  |  |  |  |  | 2 |
| *HT002* |  |  |  |  |  |  |  |  |  |
| *Butyricicoccaceae* |  | 1 |  |  |  |  |  | 1 |  |
| *Blautia* |  |  |  |  |  |  | 1 |  | 2 |
| *Megasphaera* | 1 |  |  |  |  |  |  |  | 2 |
| *Prevotella_9* |  |  |  |  |  |  | 1 |  | 1 |
| *Coprococcus* |  |  |  |  |  |  |  |  |  |
| *Sarcina* |  |  |  |  |  |  |  |  |  |
| *[Ruminococcus] gauvreauii group* |  |  |  |  |  |  |  |  |  |
| *Shuttleworthia* |  |  |  |  |  |  |  |  |  |
| *Catenibacterium* |  |  |  |  |  |  |  |  |  |
| *UCG-008* |  |  |  |  | 1 |  |  | 1 |  |
| *Phascolarctobacterium* |  |  |  |  |  |  |  |  |  |
| *Subdoligranulum* |  |  |  |  |  |  |  |  | 2 |
| *Muribaculaceae* |  |  |  |  |  |  | 1 |  |  |
| *Lachnospiraceae XPB1014 group* |  |  |  |  |  |  |  |  |  |
| *Agathobacter* |  |  |  |  |  |  |  |  |  |
| *Clostridium sensu stricto 6* |  |  |  |  |  |  |  | 1 |  |
| *Streptococcus* |  |  |  |  | 1 |  | 1 |  |  |
| *UCG-005* |  |  |  |  |  |  |  |  | 1 |
| *Prevotella* |  |  |  |  |  |  | 1 |  |  |
| *Faecalibacterium* |  |  |  |  |  |  |  |  |  |
| *Anaerovibrio* |  |  |  |  | 1 |  |  | 1 |  |
| *Prevotella_7* |  |  |  |  |  |  |  |  |  |
| *Solobacterium* |  |  |  |  |  |  |  |  |  |
| *Lachnospiraceae NK3A20 group* |  |  |  |  |  |  |  |  |  |
| *Turicibacter* |  |  |  |  |  |  |  | 1 |  |
| *Roseburia* |  |  |  |  |  |  |  |  |  |
| *Lachnospiraceae* |  |  |  | 1 |  |  | 1 |  |  |
| *[Eubacterium] hallii group* |  | 1 |  |  |  |  |  |  | 1 |
| *Intestinibacter* |  |  |  |  |  |  |  | 1 |  |
| *Dorea* |  |  |  |  |  |  |  |  |  |
| *Lachnospiraceae AC2044 group* |  | 1 |  |  |  |  |  |  |  |
| *[Eubacterium] ruminantium group* | 1 |  |  | 1 |  |  |  |  |  |
| *Oribacterium* |  |  |  |  |  |  |  |  |  |
| *Rikenellaceae RC9 gut group* |  |  |  |  |  |  |  |  |  |
| *Dialister* |  |  |  | 1 |  |  |  |  |  |
| *Marvinbryantia* | 1 |  |  |  |  |  | 1 |  |  |
| *Succinivibrio* |  |  |  |  |  |  |  |  |  |
| *Fusicatenibacter* |  |  |  |  |  |  |  |  |  |
| *Prevotellaceae NK3B31 group* |  |  |  |  |  |  |  |  |  |
| *NK4A214 group* |  |  |  |  |  |  |  |  |  |
| *Christensenellaceae R-7 group* |  |  |  |  |  |  |  |  |  |
| *Candidatus Soleaferrea* |  |  |  |  |  |  |  | 1 |  |
| *Oscillospira* |  |  |  |  |  |  |  |  |  |
| *Candidatus Saccharimonas* |  |  |  |  |  |  |  |  |  |
| *Holdemanella* |  |  |  |  | 1 |  |  |  |  |
| *Limosilactobacillus* |  | 1 |  |  |  |  |  |  |  |
| *Anaerostipes* |  |  |  |  |  |  |  |  |  |
| *Acidaminococcus* |  |  |  |  |  |  |  |  |  |
| *Butyricicoccus* |  |  |  |  |  |  |  |  |  |
| *Lachnospiraceae ND3007 group* |  |  |  |  |  |  |  |  |  |
| *UCG-002* |  |  |  |  |  |  |  |  |  |
| *Peptococcus* |  |  |  | 1 |  |  |  |  |  |
| *Ruminococcus* |  | 1 |  |  |  |  |  | 1 |  |
| *Lachnospiraceae FCS020 group* |  |  |  |  |  |  |  |  |  |
| *Romboutsia* |  |  |  |  |  |  |  |  |  |
| *Lachnospiraceae NK4A136 group* |  |  |  |  |  |  |  |  |  |
| *Prevotellaceae UCG-003* |  |  |  |  |  |  |  |  |  |
| *Methanobrevibacter* |  |  |  |  |  |  | 1 |  |  |
| *Intestinimonas* |  |  |  |  |  |  |  |  |  |
| *Incertae Sedis* |  |  |  |  |  |  |  |  |  |
| *Family XIII AD3011 group* |  |  |  |  |  |  |  |  |  |
