# Supplementary material for: The role of human–pig interactions in modulating gut microbiota, stress, and performance
Source: Porcine Health Manag. 2025 Oct 23;11:51. doi: 10.1186/s40813-025-00465-2 (PMC12548226; doi:10.1186/s40813-025-00465-2)

**Additional file 11. Differential abundance analysis of microbiome count data between the NHH and PHH groups at T2.** (A) Boxplot of microbiome multivariable associations with linear models (MaAslin2); (B) Boxplot of analysis of compositions of microbiomes with bias correction (ANCOM-BC); (C) Aldex2 plot with distribution of taxa significantly different (red dots) from the sample mean after Benjamini‒Hochberg correction; (D) Histogram plot of LDA scores (based on log10) using LEfSE. PHH = positive human handling; NHH = negative human handling; CG = control group. Significant abundant taxa are plotted at T2 (day 65) when q < 0.05.


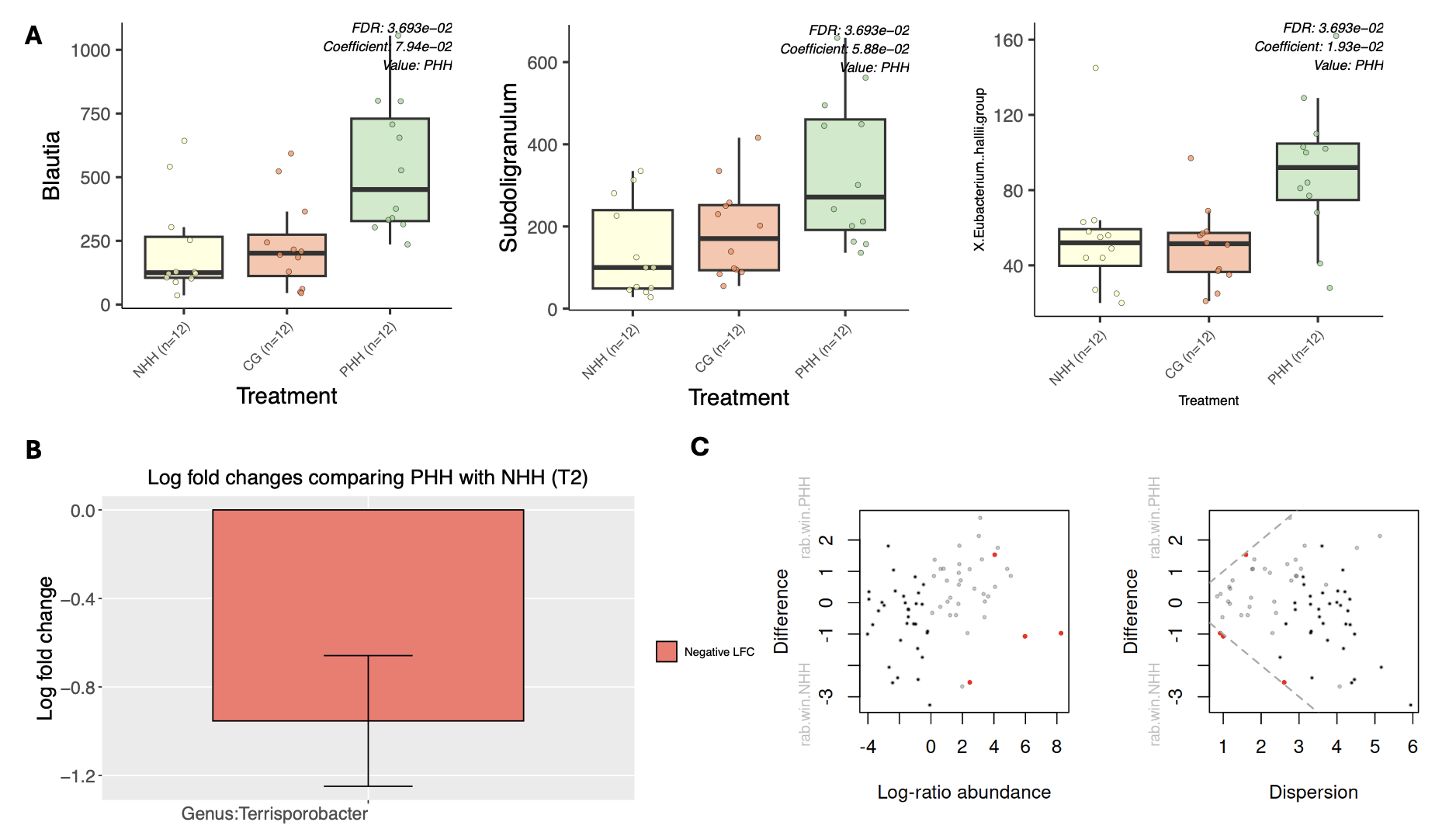


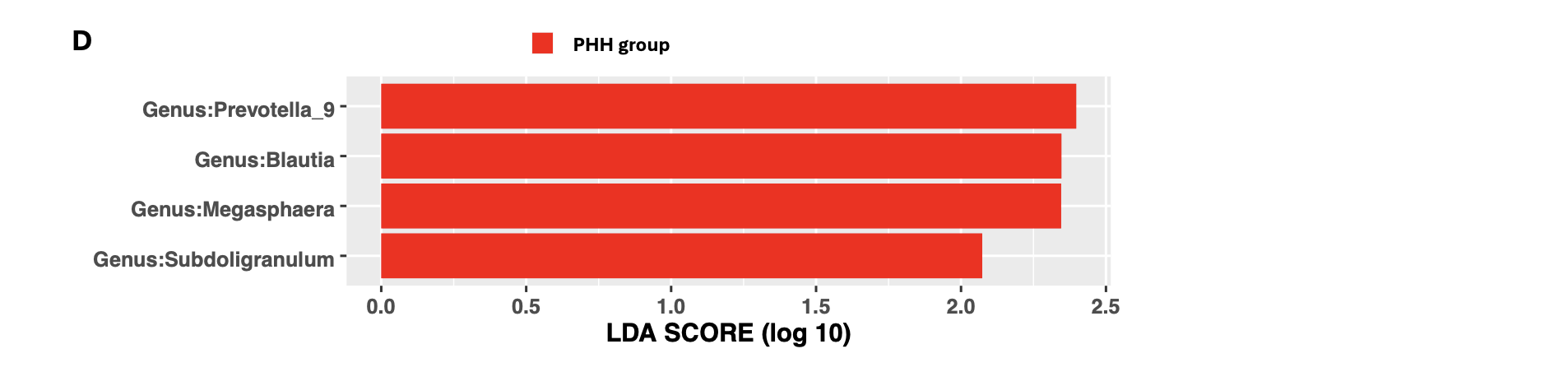

Supplement: Supplementary file 11 — Supplementary Material 11 [file 40813_2025_465_MOESM11_ESM.docx]
