# Supplementary material for: The role of human–pig interactions in modulating gut microbiota, stress, and performance
Source: Porcine Health Manag. 2025 Oct 23;11:51. doi: 10.1186/s40813-025-00465-2 (PMC12548226; doi:10.1186/s40813-025-00465-2)
Supplement: Supplementary file 12 — Supplementary Material 12 [file 40813_2025_465_MOESM12_ESM.docx]

**Additional file 12**. **Network topology for the CG, PHH, and NHH groups at T2 (day 65).** The network parameters included the number of nodes, edges, positive edge percentage, average clustering coefficient, characteristic path length, network density, heterogeneity, centralization, and the highest edge number per node. CG = control group, PHH = positive human handling; NHH = negative human handling.

| Treatment | Number of nodes | Number of edges | Positive edge percentage | Average Clustering coefficient | Characteristic Path length | Network density | Network heterogeneity | Network centralization | Highest edge number per node |
| --- | --- | --- | --- | --- | --- | --- | --- | --- | --- |
| CG | 130 | 1974 | 55.98 | 0.572 | 2.147 | 0.235 | 0.743 | 0.328 | 72 |
| PHH | 133 | 1437 | 70.01 | 0.542 | 2.756 | 0.174 | 0.868 | 0.300 | 60 |
| NHH | 89 | 402 | 98.01 | 0.468 | 3.708 | 0.112 | 0.686 | 0.190 | 25 |
