# Supplementary material for: The role of human–pig interactions in modulating gut microbiota, stress, and performance
Source: Porcine Health Manag. 2025 Oct 23;11:51. doi: 10.1186/s40813-025-00465-2 (PMC12548226; doi:10.1186/s40813-025-00465-2)

**Additional file 13**. **Box plots of node’s degrees between the CG, NHH, and PHH groups at T2 (day 65).** Nodes from all connections (ALL: positive and negative), only from positive connections (POS) and from negative connections (NEG) were considered from the whole network of microbial co‐occurrence. *P*-values < 0.05 were considered statistically significant. CG = control group; NHH = negative human handling; PHH = positive human handling.


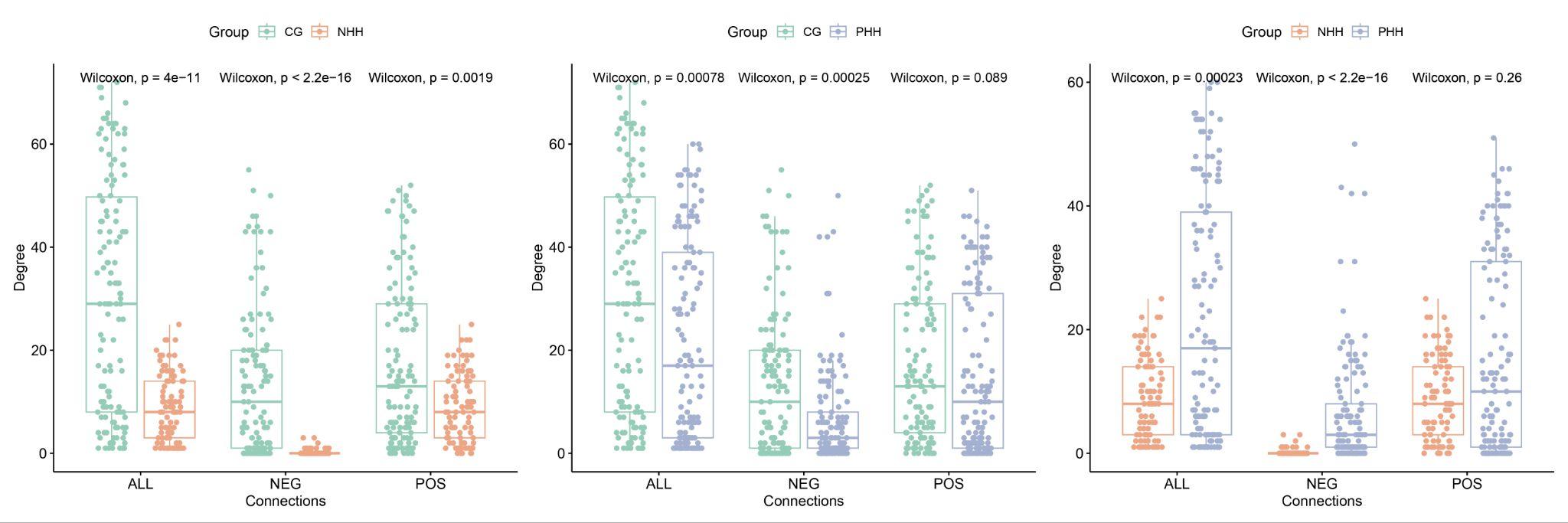

Supplement: Supplementary file 13 — Supplementary Material 13 [file 40813_2025_465_MOESM13_ESM.docx]
